# Aggregation and Degradation of White Phosphorus Mediated by N‐Heterocyclic Carbene Nickel(0) Complexes

**DOI:** 10.1002/anie.202004020

**Published:** 2020-06-03

**Authors:** Gabriele Hierlmeier, Peter Coburger, Nicolaas P. van Leest, Bas de Bruin, Robert Wolf

**Affiliations:** ^1^ Universität Regensburg Institut für Anorganische Chemie 93040 Regensburg Germany; ^2^ University of Amsterdam van 't Hoff Institute for Molecular Sciences Science Park 904 1098 XH Amsterdam The Netherlands

**Keywords:** cluster compounds, nickel, P_4_ activation, phosphorus, polyphosphides

## Abstract

The reaction of zerovalent nickel compounds with white phosphorus (P_4_) is a barely explored route to binary nickel phosphide clusters. Here, we show that coordinatively and electronically unsaturated N‐heterocyclic carbene (NHC) nickel(0) complexes afford unusual cluster compounds with P_1_, P_3_, P_5_ and P_8_ units. Using [Ni(IMes)_2_] [IMes=1,3‐bis(2,4,6‐trimethylphenyl)imidazolin‐2‐ylidene], electron‐deficient Ni_3_P_4_ and Ni_3_P_6_ clusters have been isolated, which can be described as *superhypercloso* and *hypercloso* clusters according to the Wade–Mingos rules. Use of the bulkier NHC complexes [Ni(IPr)_2_] or [(IPr)Ni(η^6^‐toluene)] [IPr=1,3‐bis(2,6‐diisopropylphenyl)imidazolin‐2‐ylidene] affords a *closo*‐Ni_3_P_8_ cluster. Inverse‐sandwich complexes [(NHC)_2_Ni_2_P_5_] (NHC=IMes, IPr) with an aromatic *cyclo*‐P_5_^−^ ligand were identified as additional products.

## Graphical abstract

**Active nickel**: Activation of white phosphorus (P_4_) by N‐heterocyclic carbene nickel(0) complexes affords binary nickel phosphorus cluster compounds. Both the degradation of P_4_ to P_1_ and P_3_ units and the aggregation to P_5_ and P_8_ moieties has been observed. The structures of the resulting Ni_3_P_4_, Ni_3_P_6_, and Ni_3_P_8_ clusters can be understood with the Wade–Mingos electron‐counting rules.

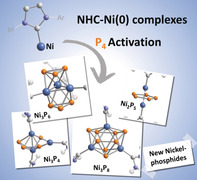

Reactions of transition metal complexes with white phosphorus present a powerful strategy to access binary metal phosphide frameworks, and the structural motifs of the resulting compounds are highly diverse.^1^, ^2^ On the one hand, degradation of P_4_ to products containing one to four phosphorus atoms is of tremendous industrial relevance, in order to improve the processes used in the production of organophosphorus compounds.^3^ On the other hand, the aggregation of P_4_ to polyphosphorus compounds with five or more phosphorus atoms is essential for understanding the structure and bonding in metal phosphides.^4^

The use of nickel as a metal for P_4_ activation may result in unique nickel phosphide clusters. Besides a few reactions of P_4_ with Ni^II^ species, for example, the formation of the sandwich compound [{(triphos)Ni}_2_(μ_2_,η^3:3^‐*cyclo*‐P_3_)](BF_4_)_2_ (triphos=Me(CH_2_CH_2_PPh_2_)_3_),^5^ known examples typically involve Ni in the +I oxidation state. Cyclopentadienyl‐substituted Ni^I^ radicals are particularly versatile, as the outcome of photolysis or thermolysis reactions of nickel complexes of the type [Cp^*x*^Ni(CO)_2_]_2_ with P_4_ is highly dependent on the size of the Cp ligand used.^6^ Relatively small cyclopentadienyl ligands such as Cp*, Cp′′ (1,3‐*t*Bu_2_C_5_H_3_), or Cp′′′ (1,2,4‐*t*Bu_3_C_5_H_2_) lead to the tetranuclear heterocubane clusters [{Cp*Ni}_3_(μ_3_,η^2:2:2^‐P_4_)(μ_3_‐P)] and [{Cp^R^Ni(μ_3_‐P)}_4_] (Cp^R^=Cp*, Cp′′], and the sandwich complex [Cp^R^Ni(η^3^‐P_3_)] (Cp^R^=Cp*, Cp′′′), whereas a trigonal‐prismatic structure [{Cp^*i*Pr^Ni}_2_(μ_2_,η^3:3^‐P_4_)] (Cp^*i*Pr^=1,2,3,4‐*i*Pr_4_C_5_H) is accessed by using a superbulky tetraisopropylcyclopentadienyl ligand. Our group recently showed that [CpNi(NHC)] (NHC=IMes, IPr) radicals can selectively activate P_4_ to afford μ_2_,η^1:1^‐P_4_ butterfly complexes.^7^

In contrast to Ni^I^ compounds, only a few examples of P_4_ activation using Ni^0^ sources have been reported (Figure 1).^8^, ^9^, ^10^ In seminal work dating back to 1979, Sacconi and co‐workers reported the formation of the complex [(*κ* ^3^‐P,P,P‐NP_3_)Ni(η^1^‐P_4_)] (**A**, NP_3_=tris(2‐diphenylphosphinoethyl)amine) containing an intact, end‐on coordinated P_4_ tetrahedron.^8^ Moreover, Le Floch and Mézailles reported on the use of [Ni(cod)_2_] (cod=1,4‐cycloocta‐1,5‐diene) for the synthesis of nickel phosphide nanoparticles.^9^ More recently, the group of Radius reported the synthesis of the butterfly compound [{Ni(Im*i*Pr_2_)_2_}_2_(μ,η^2:2^‐P_2_)] (**C**, Im*i*Pr_2_=1,3‐bis(isopropyl)imidazolin‐2‐ylidene) by reaction of cod‐stabilised Ni(Im*i*Pr_2_)_2_ fragments with P_4_.^10^ While these examples demonstrate both the coordination and degradation of P_4_ by 14 valence electron (VE) and 18 VE Ni^0^ compounds, examples of P_4_ aggregation using Ni^0^ appear to be unknown, despite an unsuccessful attempt to synthesise a sandwich complex containing a pentaphosphacyclopentadienide ligand *cyclo*‐P_5_^−^ by Miluykov, Hey‐Hawkins and co‐workers.^11^

**Figure 1:**
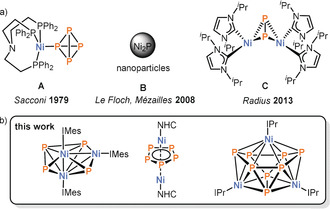
a) Overview of products resulting from P_4_ activation using Ni^0^ sources;^8^, ^9^, ^10^ b) P_4_ activation and aggregation products described herein.

Building on our previous work on P_4_ activation with N‐heterocyclic carbene (NHC) nickel(I) complexes,^7^, ^12^ we recently became interested in studying the reactivity of related Ni^0^ complexes. NHC complexes seemed promising because they can be stabilised by various labile ligands, for example, the carbenes themselves, alkenes, and arenes. After synthesising a range of known NHC compounds, including the bis(carbene) complexes [Ni(NHC)_2_] (NHC=IMes, IPr),^13^ trimethylvinylsilane complexes [(NHC)Ni(η^2^‐H_2_C=CHSiMe_3_)_2_]^14^ (NHC=IMes, IPr) and the toluene complex [(IPr)Ni(η^6^‐toluene)],^15^ we proceeded to systematically study the reactivity of these compounds toward P_4_. Reactions of [(NHC)Ni(η^2^‐H_2_C=CHSiMe_3_)_2_] (NHC=IMes, IPr) with different amounts of P_4_ afforded black, insoluble material that was not characterised any further. We next turned our attention from nickel complexes comprising labile alkene ligands to the less reactive [Ni(IMes)_2_]. Gratifyingly, the ^31^P{^1^H} NMR spectrum of the reaction of [Ni(IMes)_2_] with P_4_ (0.5 equivalents) in toluene suggested formation of a major product, characterised by two main signals in a 1:1 ratio (Scheme 1). A single‐crystal X‐ray diffraction (XRD) study of large block‐shaped crystals grown from toluene revealed the formation of the trinuclear nickel phosphorus cluster [(IMes)_3_Ni_3_P_4_] (**1**) (Figure 2).

**Figure 2:**
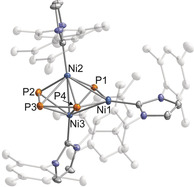
Molecular structure of **1** in the solid state. Thermal ellipsoids are set at 50 % probability level. Hydrogen atoms and solvent molecules are omitted for clarity. Selected bond lengths [Å] and angles [°]: Ni1−Ni2 2.7533(3), Ni2−Ni3 2.3720(3), Ni3−Ni1 2.6528(3), Ni1−P1 2.1045(4), Ni2−P1 2.1739(4), Ni3−P1 2.1720(4), P2−P3 2.1671(5), P3−P4 2.1754(5); Ni3‐Ni2‐Ni1 61.815(9), Ni2‐Ni3‐Ni1 66.177(9), Ni3‐Ni1‐Ni2 52.009(8), P2‐P3‐P4 106.89(2), Ni1‐P4‐P3 133.21(2), P1‐Ni1‐P4 99.288(16).

**Scheme 1:**
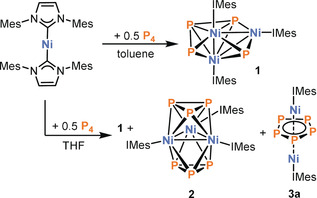
Reactivity of [Ni(IMes)_2_] toward P_4_.

The molecular structure of **1** is reminiscent of the distorted kite‐like *cyclo*‐P_4_ complex [(Cp′Fe)_2_(μ‐P_4_)] reported by Walter and co‐workers.^16^ However, **1** can be described as a bicapped trigonal bipyramid featuring a Ni_3_ triangle with one short Ni2−Ni3 bond (2.3720(3) Å) and two long nickel–nickel bonds (Ni1−Ni2: 2.7533(3) Å and Ni1−Ni3: 2.6528(3) Å). Ni_3_ triangles are a common structure motif, for example, in carbonyl‐ or phosphine‐stabilised clusters.^17^ The Ni_3_ triangle is capped by two phosphorus atoms P1 and P4. The P4 atom is part of a P_3_‐chain with P−P bond lengths of 2.1671(5) (P2−P3) and 2.1754(5) Å (P3−P4), which are in the range commonly observed for P−P single bonds. Notably, the P_4_ plane and the Ni_3_ plane are almost perpendicular with a plane twist angle of 89.6°.

Compound **1** can be isolated in pure form as a black crystalline solid in 20 % yield. As expected from analysis of the initial reaction mixture, ^31^P{^1^H} NMR measurements of pure **1** dissolved in C_6_D_6_ revealed two signals at chemical shifts of 463.1 ppm (P1/P4) and 105.6 ppm (P2/P3, averaged *J*_PP_=67.0 Hz), which are assigned to **1**. Notably, the observation of just two ^31^P{^1^H} NMR resonances is in apparent contrast with the presence of four distinct P atom positions in the solid‐state XRD structure of **1**. An additional minor signal is observed at 134.0 ppm. This signal is assigned to an unidentified species, which may be an isomer of **1**. A variable temperature (VT) NMR study showed that the integral ratio of signal P1/P4 to P2/P3 remains constant at 1:1, whereas the intensity of the signal at 134.0 ppm increases with higher temperatures and disappears upon cooling the solution to 283 K (see the Supporting Information for spectra). In order to understand this dynamic behaviour, DFT calculations were performed on a truncated model compound, where the mesityl substituents at the NHC moieties were replaced by phenyl groups. The calculations reproduce the asymmetric molecular structure of **1**, but also reveal an isoenergetic isomer (Δ*E*=−0.3 kcal mol^−1^) with a more symmetrical Ni_3_P_4_ core (see the Supporting Information for details). The fluxional behaviour observed by NMR spectroscopy can presumably be attributed to an exchange process between P1/P4 and P2/P3, which proceeds via this symmetrical isomer or a symmetrical transition state with a low energy (Δ*E*=2.6 kcal mol^−1^). The ^1^H NMR spectra are in good agreement with these findings, exhibiting three different signal sets for the IMes ligand and similar thermal dependence of the integral ratios.

Analysis of **1** by liquid field ionisation desorption mass spectrometry (LIFDI‐MS) revealed a molecular ion peak at *m*/*z=*1212.2952 in good agreement with the calculated molecular ion peak (1212.2784). The cyclic voltammogram of **1** (THF/[nBu_4_N]PF_6_, Figure S18, Supporting Information) features two reversible redox events at *E*_1/2_=−1.07 and −2.76 V (vs. Fc/Fc^+^), which may be assigned to the reversible oxidation and reduction of the complex, respectively.

The bonding situation in **1** was analysed by means of localised orbitals. In particular, intrinsic bond orbitals (IBO) were constructed starting from a BP86/def2‐TZVP wavefunction. Looking at the composition of those orbitals, six filled orbitals involving multicentre bonds between the Ni and P atoms could be identified along with a 3d^10^ configuration for each Ni atom (see the Supporting Information for a depiction). This is consistent with classical electron‐counting rules.^18^ Thus, the cluster may be defined as a *superhypercloso*‐cluster (12=2(*n*−1), *n=*7, number of cluster atoms).

The reaction of [Ni(IMes)_2_] with P_4_ is significantly less selective when THF is used as a solvent instead of toluene. Besides **1**, two other products formed could be identified by ^31^P{^1^H} NMR spectroscopy and X‐ray crystallography. After work‐up, brown crystals of the trinuclear cluster [(IMes)_3_Ni_3_P_6_] (**2**) were obtained from *n*‐hexane (Figure 3). Structural analysis of **2** reveals a distorted tricapped trigonal prism (or, equivalently, two facial Ni_3_P_3_ octahedra sharing a common Ni_3_ face). Notably, compounds featuring pnictogen (P, As) prisms with iron or cobalt are usually stabilised by anionic cyclopentadienyl ligands.^19^ Similar to **1**, an unsymmetrical Ni_3_‐triangle is observed (Ni1−Ni2 2.4835(3) Å, Ni1−Ni3 2.4882(3) Å, Ni2−Ni3 2.6429(3) Å). The P−P bond lengths range from 2.2055(4) to 2.2700(4) Å consistent with P−P single bonds. The ^31^P{^1^H} NMR spectrum in C_6_D_6_ shows a broad resonance at −8.6 ppm. The bonding situation in **2** was analysed similarly to that in cluster **1**. In accordance with electron‐counting rules, nine doubly occupied orbitals of multicentre bonds between the cluster atoms were identified (see the Supporting Information for a depiction). Thus, due to its closed deltahedral structure (distorted tricapped trigonal prism) and fulfilment of the 2*n* cluster electron rule (*n*=9), **2** can be described as a 9‐vertex *hypercloso*‐cluster. Additionally, a 3d^10^ configuration for each Ni atom in **2** could be derived from the analysis of the IBO (see the Supporting Information for details).

**Figure 3:**
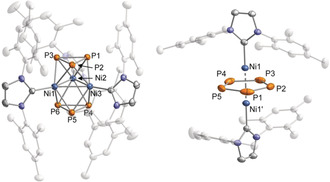
Molecular structure of **2** (left) and **3 a** (right) in the solid state. Thermal ellipsoids are set at 50 % probability level. Hydrogen atoms, solvent molecules and disorder in the P_5_ ring (**3 a**) are omitted for clarity. Selected bond lengths [Å] and angles [°] for **2**: Ni1−Ni2 2.4834(3), Ni1−Ni3 2.4883(3), Ni2−Ni3 2.6432(3), P1−P2 2.2087(5), P2−P3 2.2698(5), P1−P3 2.2156(5), P4−P5 2.2116(5), P5−P6 2.2822(5), P4−P6 2.2049(5); Ni2‐Ni1‐Ni3 64.233(10), Ni1‐Ni2‐Ni3 57.974(9), Ni1‐Ni3‐Ni2 57.793(9), P2‐P1‐P3 61.729(16), P1‐P2‐P3 59.285(16), P1‐P3‐P2 58.985(16), P6‐P4‐P5 62.226(16), P4‐P5‐P6 58.744(16), P4‐P6‐P5 59.030(16); **3 a**: Ni1−Ni1′ 2.6339(13), P1−P2 2.182(8), P2−P3 2.194(7), P3−P4 2.205(8) P4−P5 2.211(9), P5−P1 2.207(7); P2‐P1‐P5 108.2(2), P1‐P2‐P3 108.7(2), P2‐P3‐P4 107.6(3), P3‐P4‐P5 108.1(3), P1‐P5‐P4 107.4(3).

Moreover, we were able to identify [(IMes)_2_Ni_2_P_5_] (**3 a**) as a side product. This compound co‐crystallises with **2** from the mother liquor of the reaction mixture of [Ni(IMes)_2_] with P_4_. Structural analysis of crystals of the composition [(IMes)_3_Ni_3_P_6_]⋅[(IMes)_2_Ni_2_P_5_] (**2**⋅**3 a**) revealed that compound **3 a** features a dinuclear inverse sandwich structure in the solid state with a bridging *cyclo*‐P_5_^−^ ligand (Figure 3). The Ni1−Ni1′ distance is 2.6339(13) Å and the P−P bond lengths range from 2.182(8) to 2.211(9) Å, which is in the common range observed for dinuclear 3d transition metal complexes with bridging *cyclo*‐P_5_^−^ ligands.^20^, ^21^ The pentaphosphacyclopentadienyl ligand is frequently observed in transition metal mediated P_4_ activation.^1^ However, most complexes comprising such a *cyclo*‐P_5_^−^ ligand feature group 8 metals and there are only a few examples of other transition metal complexes.^21^ Furthermore, all known *cyclo*‐P_5_^−^ complexes additionally contain cyclopentadienyl ligands, while complex **3 a** is stabilised by an L‐type ligand.

Having established the ability of [Ni(IMes)_2_] to act as a precursor to interesting Ni/P clusters, we proceeded with performing the analogous reactions using the bulkier carbene complex [Ni(IPr)_2_] in order to examine if there is any difference in product distribution (Scheme 2). And, indeed, in contrast to observations made using [Ni(IMes)_2_], ^31^P{^1^H} NMR spectroscopy revealed no resonances. Nevertheless, the ^1^H NMR spectrum clearly showed the formation of free IPr and one new distinct diamagnetic IPr environment.

**Scheme 2:**
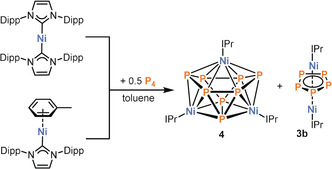
Reactivity of [Ni(IPr)_2_] and [(IPr)Ni(η^6^‐toluene)] toward P_4_.

Furthermore, a single‐crystal X‐ray diffraction study on crystals grown from toluene revealed the formation of [(IPr)_3_Ni_3_P_8_] (**4**), an 11‐vertex *closo*‐cluster with 24 cluster electrons, adopting an octadecahedral geometry similar to the undecaborate anion [B_11_H_11_]^2−^ (Figure 4).^22^ The homoquadricyclane‐like P_8_ framework is reminiscent of the P_8_ subunits in Hittorf's phosphorus and can be seen as a formal insertion product of Ni in one of the P−P bonds of such a subunit.^23^ Nevertheless, to the best of our knowledge, this is the first example of such a P_8_ framework in an isolated molecular compound.^4^ The structure of compound **4** again comprises three Ni atoms, but the Ni⋅⋅⋅Ni distances are significantly longer than in complexes **1** and **2** [Ni1⋅⋅⋅Ni2 3.3246(18) Å and Ni2⋅⋅⋅Ni2′ 3.636(2) Å]. Ni1 is coordinated by six P atoms (P1, P1′, P2, P2′, P3, P3′) and Ni2/Ni2′ are coordinated by five P atoms (P1, P2, P3, P4 P4′ for Ni2 and P1′, P2′, P3′, P4, P4′ for Ni2′). The P_8_‐framework contains short P−P bonds ranging from 2.201(3) to 2.288(3) Å (P1−P3, P1−P2, P2−P3′), and long P−P bonds with bond lengths from 2.349(4) to 2.459(3) Å (P4−P4′, P3−P4′, P2−P4).

**Figure 4:**
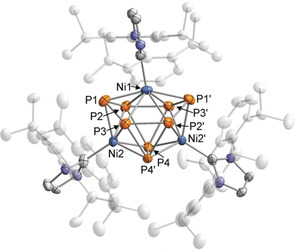
Molecular structure of **4** in the solid state. Thermal ellipsoids are set at 50 % probability level. Hydrogen atoms, solvent molecules and disorder in the IPr ligand are omitted for clarity. Selected bond lengths [Å] and angles [°]: Ni1⋅⋅⋅Ni2 3.3246(18), Ni2⋅⋅⋅Ni2′ 3.636(2), P1−P2 2.205(3), P1−P3 2.201(3), P2−P3′ 2.288(3), P2−P4 2.459(3), P3−P4′ 2.434(3), P4−P4′ 2.349(4); Ni2‐Ni1‐Ni1′ 66.31(5), Ni1‐Ni2‐Ni2′ 56.85(3), P3‐P1‐P2 103.21(11), P3′‐P2‐P4 61.57(8), P2′‐P3‐P4′ 62.67(9), P3′‐P4‐P2 55.77(8).

^1^H and ^13^C{^1^H} NMR spectra of crystals of **4** dissolved in C_6_D_6_ showed only one set of IPr signals despite the presence of two distinct IPr environments in the solid‐state structure. This evidence for fluxionality in solution was further confirmed by variable‐temperature ^31^P{^1^H} NMR spectroscopy (Figure 5). Coincidentally, the spectrum recorded at room temperature exhibits an extremely broad signal that could not be resolved. However, heating up the solution results in one broad resonance, whereas cooling the solution to 193 K afforded three signals with an integral ratio of 4:2:2, at chemical shifts of −136.2 (P2, P2′, P3, P3′), 97.0 (P1, P1′ or P4, P4′) and 124.6 ppm (P1, P1′ or P4, P4′), which is in agreement with the presence of three different P environments as suggested by the crystallographic study. Even at 193 K, the couplings could not be resolved completely.

**Figure 5:**
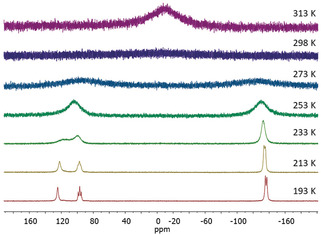
VT ^31^P{^1^H} NMR spectra of [(IPr)_3_Ni_3_P_8_] (**3**) in [D_8_]toluene.^24^

Unfortunately, separation of free IPr from compound **4** proved to be challenging. The use of [(IPr)Ni(η^6^‐toluene)] as an attractive precursor was therefore pursued and led to the isolation of pure **4** as a dark green powder in 41 % yield. The cyclic voltammogram of **4** (THF/[nBu_4_N]PF_6_, Figure S20) shows one reversible oxidation wave at *E*_1/2_=−0.76 V (vs. Fc/Fc^+^). Analysis of the IBO reveals 12 orbitals that involve bonding between the cluster atoms again being in accordance with established electron‐counting rules. Thus **4** obeys the 2(*n*+1) (*n=*11) electron count rule of a 11‐vertex *closo*‐cluster (see the Supporting Information for a depiction of the IBO). The same analysis additionally allows for the assignment of a d^8^‐configuration for the Ni1 atom and d^10^‐configurations for Ni2/Ni2′.

Apart from **4**, the reaction of [(IPr)Ni(η^6^‐toluene)] with P_4_ also affords green crystals of [(IPr)_2_Ni_2_(μ‐P_5_)] (**3 b**), which were obtained from the *n*‐hexane washing solution and identified by X‐ray crystallography. Complex **3 b** is isostructural with **3 a** and features similar Ni−Ni and P−P bond lengths (see the Supporting Information for further details).

The electronic structure of a slightly truncated model complex **3′** ([(IPh)_2_Ni_2_P_5_], IPh=1,3‐diphenylimidazolin‐2‐ylidene) was calculated at the TPSSh/IGLO‐III (CP(PPP) on Ni) level of theory.^25^ This method was chosen since it has proven to yield reliable results for the calculation of magnetic properties. Significant interactions between the Ni atoms (Mayer bond order: 0.8) as well as the Ni atoms and the aromatic P_5_ ring were found (Mayer bond order: 0.5). The X‐band EPR spectrum of **3 b** (Figure 6) recorded in a toluene glass at 20 K reveals an axial signal pattern for an S=1/2 system showing hyperfine interactions with all five phosphorus atoms. A satisfactory simulation of the experimental spectrum was obtained assuming hyperfine interactions with five equivalent phosphorus atoms (*g*_11_=*g*_22_=2.186 (2.11), *g*_33_=1.987 (2.01), *A* ^31P^_33_=30.0 MHz (27.5 MHz, averaged value, DFT‐calculated values of **3′** in parentheses; see the Supporting Information). Given the good agreement between the measured and DFT calculated EPR parameters, the calculated and the true electronic structure should resemble each other closely. Thus, according to our DFT calculations, the spin density in 3’ is evenly distributed between the Ni atoms (Figure 6).

**Figure 6:**
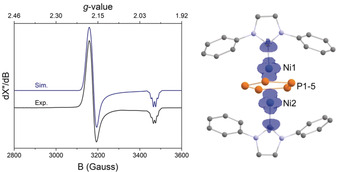
Left: Experimental and simulated X‐band EPR spectrum of **3 b** in a toluene glass at 20 K. Freq. 9.6508 GHz, 0.6325 mW, 20 K, mod. 4.000 Gauss; g‐tensor parameters obtained from simulations and DFT calculations for **3′** are: *g*_11_=*g*_22_=2.186 (2.11), *g*_33_=1.987 (2.01); DFT‐calculated values are given in parentheses. Right: spin density (blue) of [(IPh)_2_Ni_2_P_5_] (**3′**) calculated by DFT.

To conclude, reactions of N‐heterocyclic carbene nickel(0) complexes with P_4_ afford unprecedented nickel phosphorus clusters. These reactions clearly show an impact of the size of the NHC ligand on the products obtained. Upon increasing the steric demand from I*i*Pr_2_ to IMes, di‐ and trinuclear complexes with Ni_3_P_4_ (**1**), Ni_3_P_6_ (**2**) cores as well as Ni_2_P_5_ (**3 a**) were obtained. Notably, **3 a** represents the first nickel pentaphosphacyclopentadienyl complex. The bulky NHC IPr again changes the outcome of the reaction to afford a Ni_3_P_8_ (**4**) *closo*‐cluster with a novel homoquadricyclane‐like P_8_ framework. Bulky substituents on the NHC ligands presumably facilitate the formation of monocarbene nickel fragments observed in the molecular structures of **1**–**4**. However, the mechanism of formation of these products is obviously complex, and the details of the initial P_4_ activation process and the subsequent transformations of the resulting intermediates must be revealed by further studies. Moreover, we are currently investigating the use of **1**–**4** as single‐source precursors for the preparation of nickel phosphides as electrocatalysts for hydrogen evolution.^26^

## Conflict of interest

The authors declare no conflict of interest.

## Supporting information

As a service to our authors and readers, this journal provides supporting information supplied by the authors. Such materials are peer reviewed and may be re‐organized for online delivery, but are not copy‐edited or typeset. Technical support issues arising from supporting information (other than missing files) should be addressed to the authors.

Supplementary
