# Supplementary material for: Aggregation and Degradation of White Phosphorus Mediated by N‐Heterocyclic Carbene Nickel(0) Complexes
Source: Angew Chem Int Ed Engl. 2020 Jun 3;59(33):14148–53. doi: 10.1002/anie.202004020 (PMC7496536; doi:10.1002/anie.202004020)
Supplement: Supplementary file 1 — Supplementary [file ANIE-59-14148-s001.pdf]

Angewandte Chemie
Eine Zeitschrift der Gesellschaft Deutscher Chemiker

Supporting Information

Aggregation and Degradation of White Phosphorus Mediated by N-Heterocyclic Carbene Nickel(0) Complexes

*Gabriele Hierlmeier, Peter Coburger, Nicolaas P. van Leest, Bas de Bruin, and Robert Wolf**

anie_202004020_sm_miscellaneous_information.pdf

Supporting Information

The Supporting Information contains:

1 Synthetic Procedures 2

1.1 General Synthetic Methods 2

1.2 General Analytical Techniques 2

1.3 Synthesis of Compounds 3

2 NMR Spectra 7

3 UV-Vis Spectra 15

4 Cyclic Voltammetry 16

5 EPR Spectroscopy 18

5.1 General Method 18

5.2 Experimental and Simulated EPR Spectra 18

6 Single Crystal X-ray Diffraction Data 21

7 Quantum Chemical Calculations 25

1 Synthetic Procedures

1.1 General Synthetic Methods

All reactions and product manipulations were carried out in flame-dried glassware under an inert atmosphere of argon using standard Schlenk-line or glovebox techniques (maintained at <0.1 ppm H_2O and <0.1 ppm O_2). [Ni(IMes)$_2$],[1] [Ni(IPr)$_2$],[2] [Ni(IPr)(toluene)],[3] [(IMes)Ni(η^2-H_2C=CHSiMe$_3$)$_2$][4] and [(IPr)Ni(η^2-H_2C=CHSiMe$_3$)$_2$][5] were prepared according to procedures previously reported in the chemical literature.

Solvents were dried and degassed with a MBraun SPS800solvent purification system. All dry solvents except *n*-hexane and *n*-pentane were stored under argon over activated 3 Å molecular sieves in gas-tight ampules. *n*-Hexane and *n*-pentane were stored over a potassium mirror.

1.2 General Analytical Techniques

NMR spectra were recorded on Bruker Avance 300 or 400 spectrometers at 300 K unless otherwise noted and internally referenced to residual solvent resonances (^{1}H NMR: THF-d^8: 1.72 ppm, C_6D_6: 7.16 ppm, toluene-d^8: 2.08 ppm; ^{13}C{^{1}H} NMR: THF-d^8: 25.31 ppm, C_6D_6: 128.06 ppm). Chemical shifts δ are given in ppm referring to external standards of tetramethylsilane (^{1}H, ^{13}C{^{1}H}), 85% phosphorus acid (^{31}P and ^{31}P{^{1}H} spectra). ^{1}H and ^{13}C NMR signals were assigned based on 2D NMR spectra (^{1}H,^{1}H-COSY, ^{1}H,^{13}C-HSQC, ^{1}H,^{13}C-HMQC).

UV-Vis spectra were recorded on an Ocean Optics Flame Spectrometer. High resolution mass spectra were recorded by the analytical department at the University of Regensburg using a Jeol AccuTOF GCX. Elemental analysis was performed by the central analytics department of the University of Regensburg.

1.3 Synthesis of Compounds

[$(IMes)_3Ni_3P_4$] (1):

To a mixture of [$Ni(IMes)_2$] (200 mg, 0.30 mmol, 1.0 eq.) and white phosphorus (18 mg, 0.15 mmol, 0.5 eq.) was added cold (−30 °C) toluene (10 mL). The dark blue reaction mixture underwent a colour change to yellow-brown within 1 hour. The solution was stirred at ambient temperature for 18 hours. Subsequently, the solvent was removed under reduced pressure and the dark residue was dried *in vacuo*. The residue was washed with *n*-hexane (3x2 mL) and extracted with toluene (1 mL). Slow diffusion of *n*-hexane into the toluene solution yielded dark black blocks of the solvate **1·toluene**, containing 1 molecule of toluene per molecule of **1**. The crystals were used for a single crystal X-ray structure determination, elemental analysis, LIFDI-MS and UV/VIS spectroscopy.

For NMR spectroscopy, the crystals of **1·toluene** were dissolved in C_6D_6. Subsequently, the solvent was removed in order to remove toluene. The resulting brown powder was re-dissolved in C_6D_6.

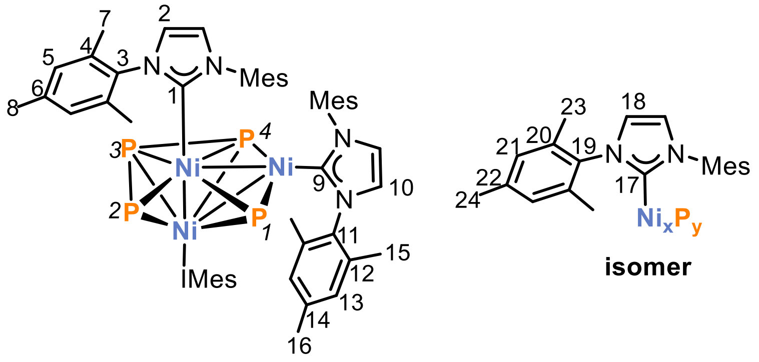

$C_{63}H_{72}N_6Ni_3P_4$, MW = 1213.29 g/mol (for dry powder sample)

$C_{70}H_{80}N_6Ni_3P_4$, MW = 1305.43 g/mol (for crystals containing 1 eq. of toluene)

Yield: 24 mg (20%)

^{1}H NMR (400 MHz, 300 K, C_6D_6) δ = 1.93 (s, 12H, C^7H), 2.09 (s, 2H, $C^{23}H$), 2.22 (s, 8H, $C^{15}H$+$C^{24}H$), 2.29 (s, 6H, C^8H), 2.47 (s, 3H, $C^{16}H$), 6.07 (s, 2H, C^2H), 6.22 (s, 0.4H, $C^{18}H$), 6.30 (s, 1H, $C^{10}H$), 6.79 (s, 0.8H, $C^{21}H$), 6.82 (s, 4H, C^5H), 7.00 (s, 2H, $C^{13}H$) ppm.

^{13}C{^{1}H} NMR (100 MHz, 300 K, C_6D_6) δ = 18.9 (s, C^{23}), 19.1 (s, C^7), 19.3 (s, C^{15}), 21.4 (s, C^{24}), 21.5 (s, C^8), 21.6 (s, C^{16}), 120.7 (s, C^{18}), 121.1 (s, C^{10}), 121.5 (s, C^2), 129.0 (s, C^5), 129.1

(s, C^{21}), 129.4 (s, C^{13}), 135.5 (s, C^{12}), 135.6 (s, C^{20}), 135.7 (s, C^{4}), 137.0 (s, C^{6}), 137.1 (s, C^{22}), 137.3 (s, C^{14}), 137.7 (s, C^{19}), 137.8 (s, C^{3}), 138.5, (s, C^{11}), 187.7 (brs, $C^{carbene(s)}$) ppm.

^{31}P{^{1}H} NMR (162 MHz, 300 K, C_6D_6) δ = 105.6 (t, J_{PP} = 67.0 Hz, P2/P3), 134.0 (brs, isomer), 463.1(brs, P1/P4) ppm.

Elemental Analysis calcd. for $C_{63}H_{72}N_6Ni_3P_4{\cdot}C_7H_8$ (**1**·toluene) C 64.41, H 6.18, N 6.44; found C 64.72, H 6.39, N 6.40.

UV/VIS (THF): λ_{max} (nm, ε_{max} /L·mol^{-1}·cm^{-1}) 300 (17700), 370 (16600), 430 (10500sh).

LIFDI-MS m/z = 1212.2952 (calc. 1212.2784).

[(IMes)$_3$Ni$_3$P$_6$] (2) and [(IMes)$_3$Ni$_3$P$_6$]·[(IMes)$_2$Ni$_2$P$_5$] (2·3a):

To a mixture of [Ni(IMes)$_2$] (100 mg, 0.15 mmol, 1.0 eq.) and white phosphorus (9 mg, 0.75 mmol, 0.5 eq.) was added cold (−30 °C) thf (5 mL). The dark blue reaction mixture underwent a colour change to yellow-brown within 5 minutes. The solution was stirred at ambient temperature for 2 hours. Subsequently, the solvent was removed under reduced pressure and the dark residue was dried *in vacuo*. The residue was extracted with *n*-hexane (3x3 mL) and concentrated to ca. 5 mL. A ^{31}P{^{1}H} NMR spectrum of the crude mixture is shown in Figure S8. Dark brown needles of **2** suitable for single crystal X-ray crystallography were obtained by leaving the saturated solution in *n*-hexane at ambient temperature overnight. These crystals were characterized by ^{1}H and ^{31}P{^{1}H} NMR spectroscopy and elemental analysis.

Dark blocks of **2**·**3a** were obtained by storing the supernatant solution at −30 °C for two weeks. These crystals were used for EPR spectroscopy (see Chapter 5).

Note that compound **2** is difficult to purify due to the formation of free IMes, which shows a similar solubility. IMes can be removed by fractional crystallization. Unfortunately, attempts to purify crude **2** by column chromatography over silica gel or aluminium oxide were unsuccessful.

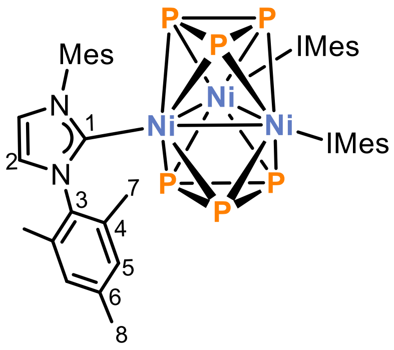

$C_{63}H_{72}N_6Ni_3P_6$, MW = 1275.23 g/mol

Yield: 6 mg (9%)

^{1}H NMR (400 MHz, 300 K, C_6D_6) δ = 2.23 (s, 18H, C^8H), 2.28 (s, 36H, C^7H), 6.87 (s, 12H, C^5H), 7.9 (s, 6h, C^2H) ppm.

A meaningful **^{13}C{^{1}H} NMR** spectrum could not be obtained due to slow decomposition of **2** in solution over several hours.

^{31}P{^{1}H} NMR (162 MHz, 300 K, C_6D_6) δ = −8.6 (brs) ppm.

Elemental Analysis calcd. C 59.34, H 5.69, N 6.59; found C 56.99, H 5.52, N 6.06. Low carbon values were found repeatedly in three independent samples.

[(IPr)$_3$Ni$_3$P$_8$] (4):

To a mixture of [(IPr)Ni(η^6-toluene)] (130 mg, 0.24 mmol, 1.0 eq.) and white phosphorus (21 mg, 0.17 mmol, 0.67 eq.) was added cold (−30 °C) toluene (50 mL). The dark red reaction mixture underwent a colour change to dark green-brown within 5 minutes. The solvent was removed and the dark residue was dried *in vacuo*. Subsequently, the solid was washed with *n*-hexane (3x5 mL) until the filtrate was colourless. The remaining dark green powder was analytically pure **4**. Crystals suitable for X-ray crystallography were grown by cooling down a saturated solution of **4** in an appropriate solvent (*n*-hexane, *n*-heptane, toluene, Et_2O) from ambient temperature to −30 °C (see Chapter 6).

The solvent of the *n*-hexane washing solution was removed *in vacuo*. The ^{1}H NMR spectrum revealed a mixture of free IPr, **4** and **3b** (see Figure S15). This sample was used for the EPR measurement described in chapter 5.

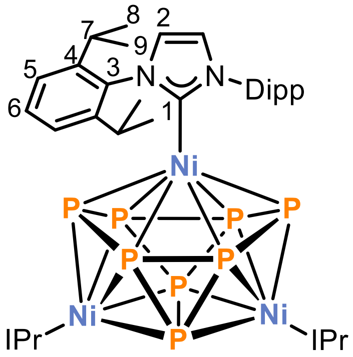

$C_{81}H_{108}N_6Ni_3P_8$, MW = 1589.67 g/mol

Yield: 53 mg (41%)

^{1}H NMR (400 MHz, 300 K, C_6D_6) δ = 1.14 (d, $^{3}J_{HH}$ = 6.8 Hz, 36H, C^8H), 1.41 (d, $^{3}J_{HH}$ = 6.7 Hz, 36H, C^9H), 3.16 (sept, $^{3}J_{HH}$ = 6.8 Hz, 12H, C^7H), 6.77 (s, 6H, C^2H), 7.13 (s, 18H, $C^{5/6}$H) ppm.

^{13}C{^{1}H} NMR (400 MHz, 300 K, C_6D_6) δ = 23.7 (s, C^9), 26.4 (s, C^8), 28.9 (s, C^7), 124.1 (s, $C^{5/6}$), 124.4 (s, C2), 128.6 (s, $C^{5/6}$), 138.0 (s, C^3), 145.8 (s, C^4) ppm.

Elemental Analysis calcd. C 61.20, H 6.85, N 5.29; found C 60.84, H 6.77, N 4.94.

UV/VIS (THF): λ_{max} (nm, ε_{max} /L·mol^{-1}·cm^{-1}) 300 (14600).

2 NMR Spectra

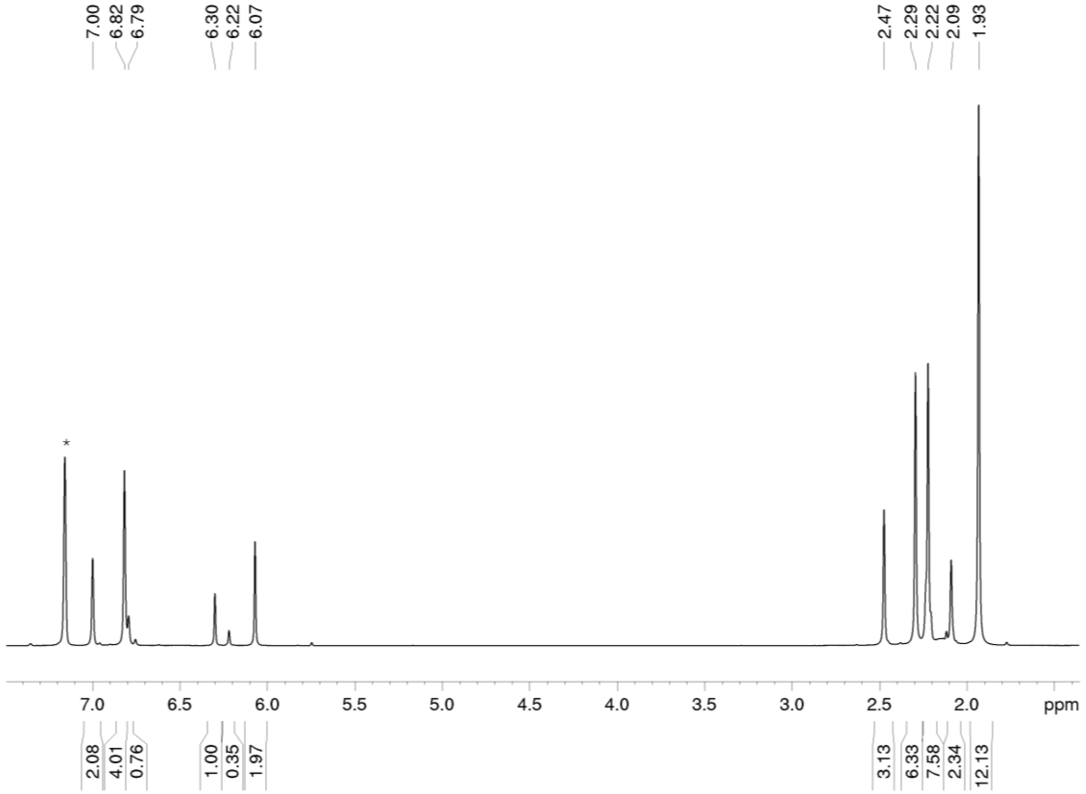

Figure S1. ^{1}H NMR spectrum (400 MHz, 300 K, C_6D_6) of **1**; *C_6D_6.

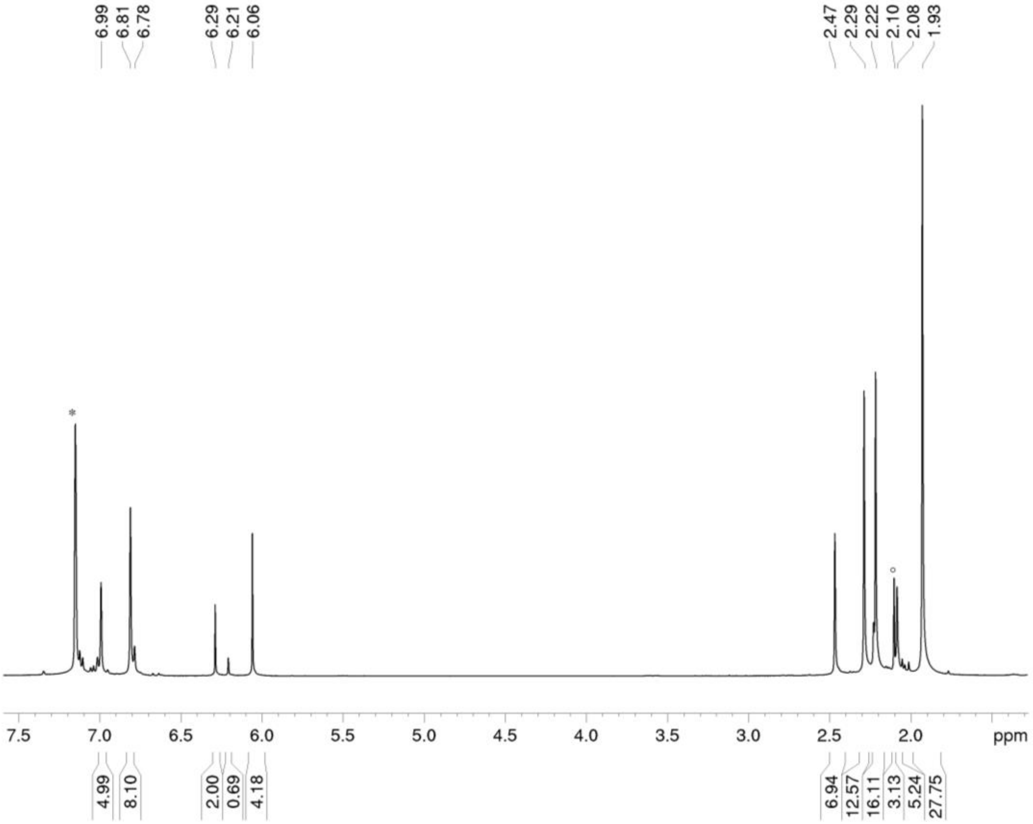

Figure S2. ^{1}H NMR spectrum (400 MHz, 300 K, C_6D_6) of **1**·toluene; *C_6D_6; °toluene-CH_3.

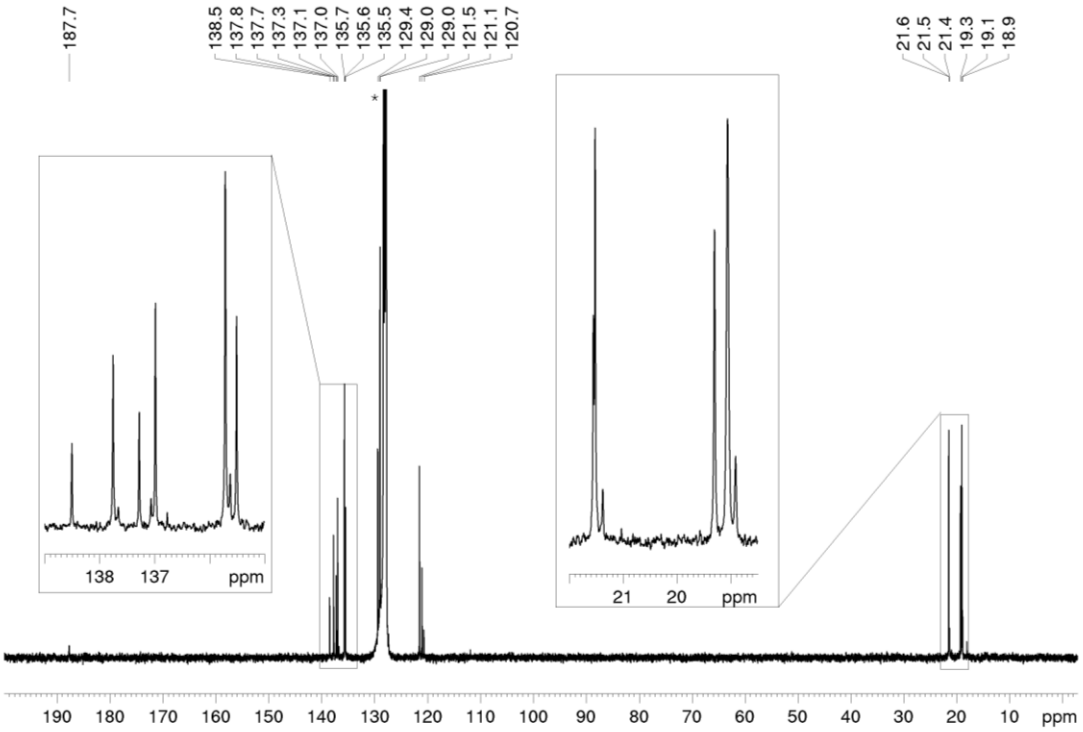

Figure S3. $^{13}C\{^1H\}$ NMR spectrum (400 MHz, 300 K, C_6D_6) of **1**; *C_6D_6.

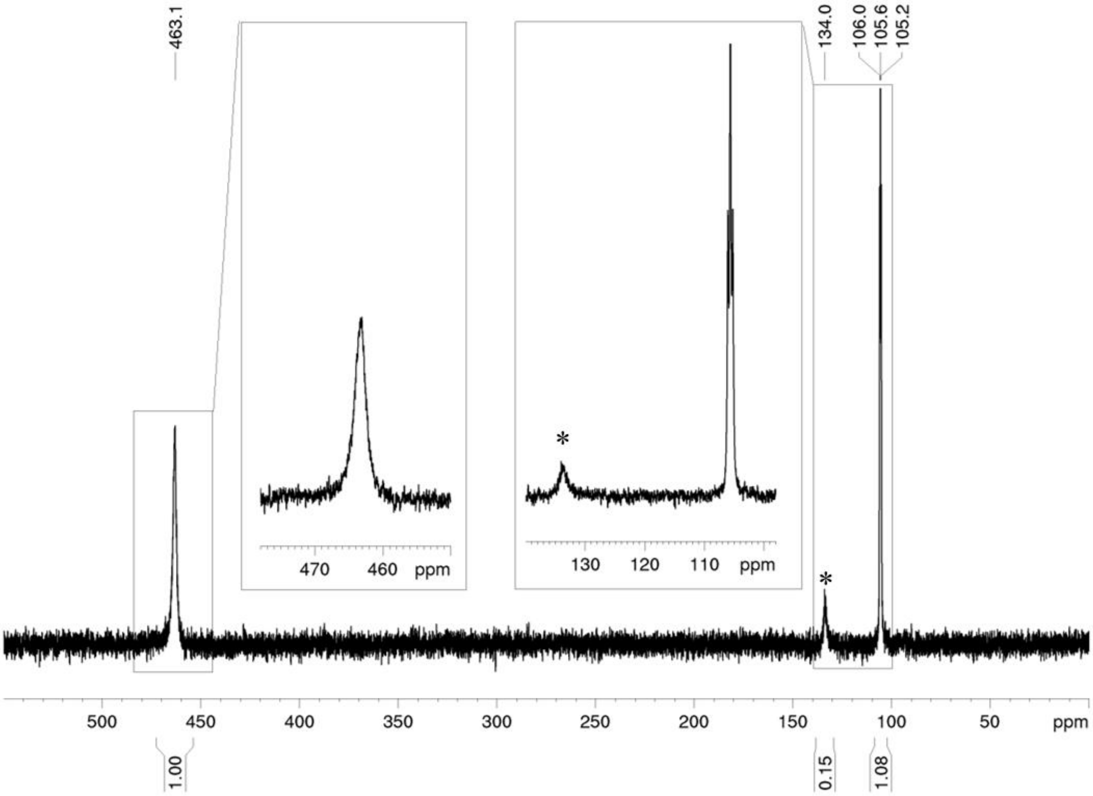

Figure S4. $^{31}P\{^1H\}$ NMR spectrum (162 MHz, 300 K, C_6D_6) of **1**; *isomer in solution.

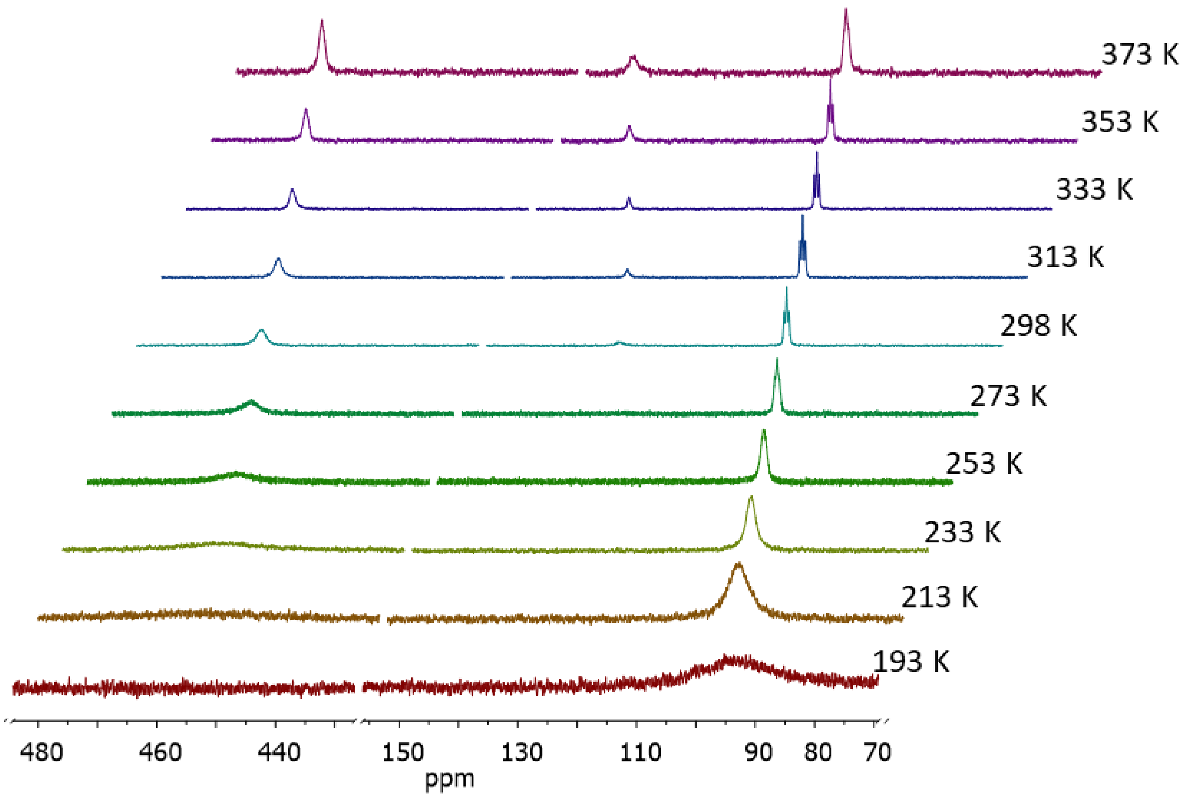

Figure S5. Variable temperature $^{31}P\{^{1}H\}$ NMR (162 MHz, 193-373 K, toluene-d^{8}) of **1**.

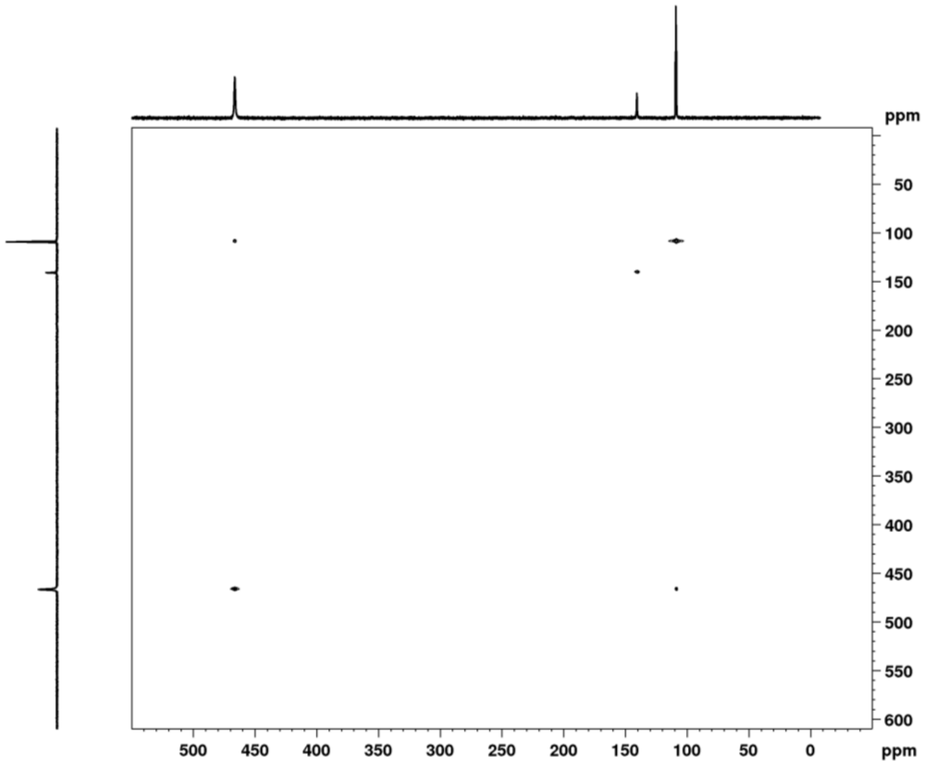

Figure S6. $^{31}P\{^{1}H\}$-$^{31}P\{^{1}H\}$ COSY NMR (162 MHz, 333 K, toluene-d^{8}) of **1**.

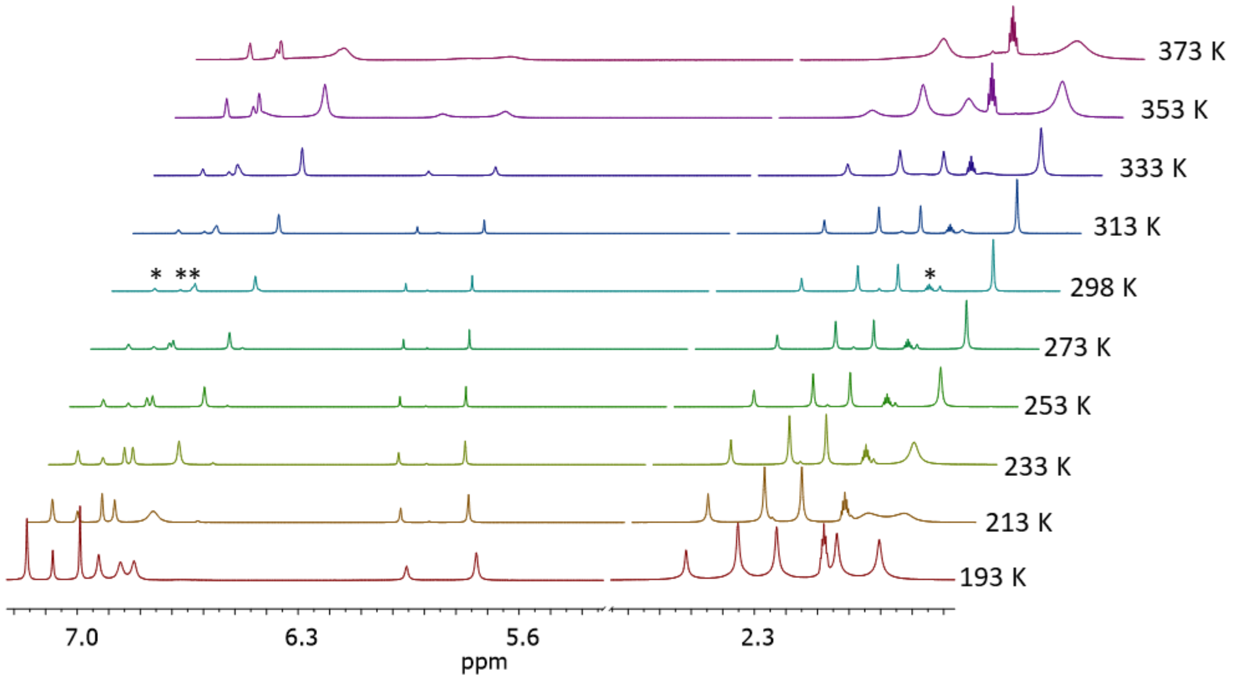

Figure S7. Variable temperature ^{1}H NMR (400 MHz, 193-373 K, toluene-d^{8}) of **1**, *toluene-d^{8}.

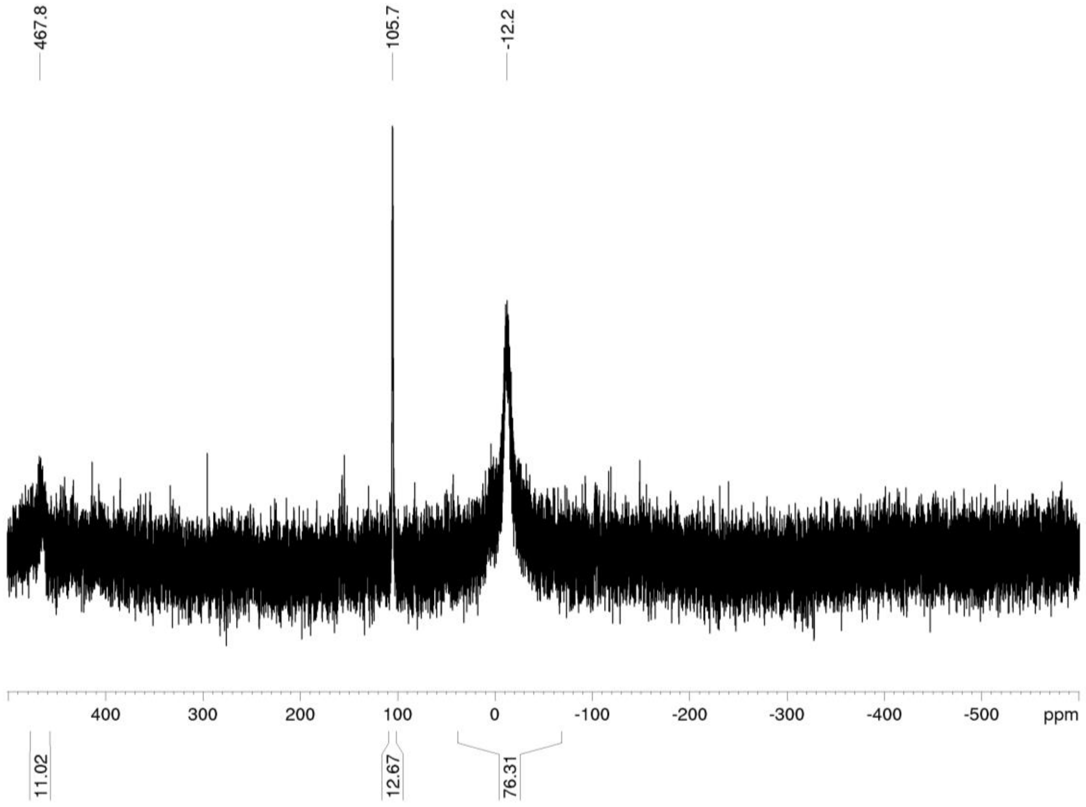

Figure S8. $^{31}P\{^{1}H\}$ NMR spectrum (162 MHz, 300 K, C_6D_6) of the reaction of [(IMes)$_2$Ni] + 0.5 eq. P_4 (in THF).

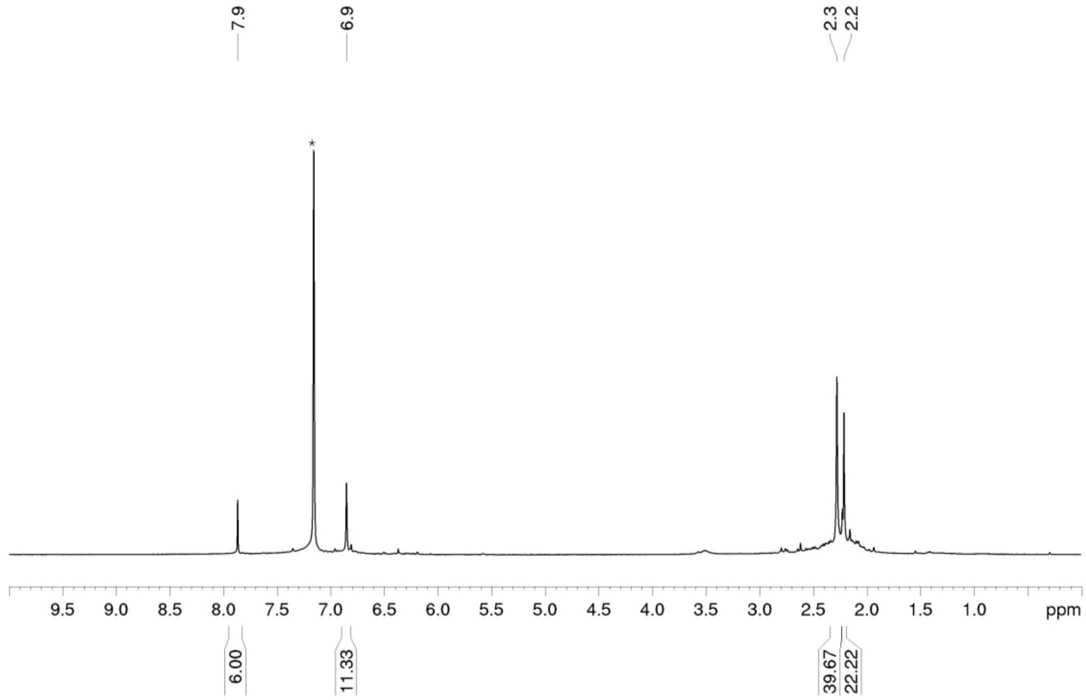

Figure S9. ^{1}H NMR spectrum (400 MHz, 300 K, C_6D_6) of **2**, *C_6D_6.

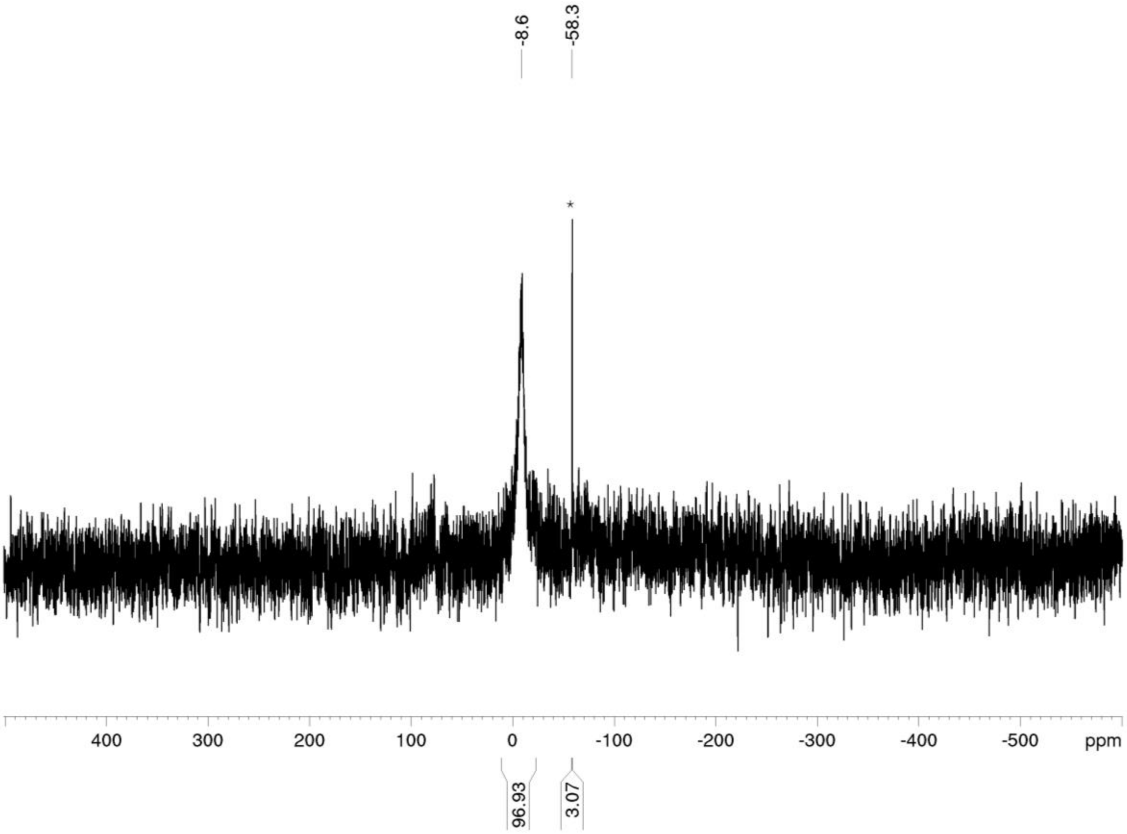

Figure S10. $^{31}P\{^{1}H\}$ NMR spectrum (162 MHz, 300 K, C_6D_6) of **2**, * unknown impurity.

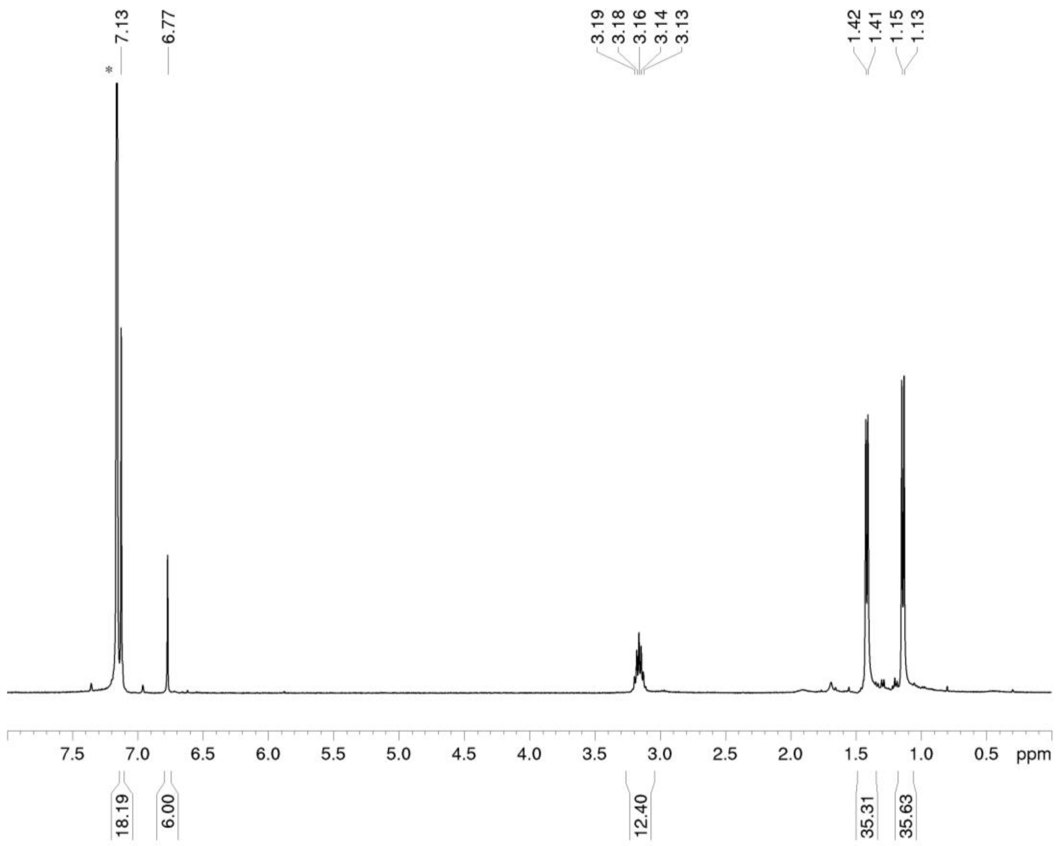

Figure S11. ^{1}H NMR spectrum (400 MHz, 300 K, C_6D_6) of **4**, *C_6D_6.

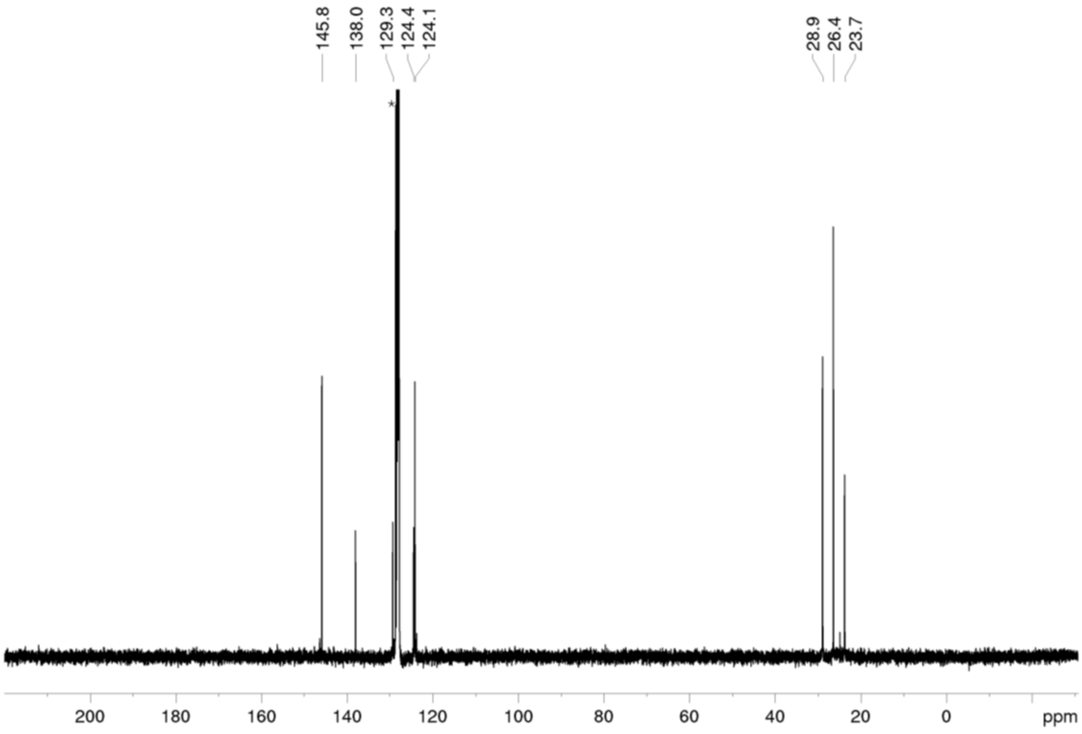

Figure S12. $^{13}C\{^1H\}$ NMR spectrum (400 MHz, 300 K, C_6D_6) of **4**; *C_6D_6.

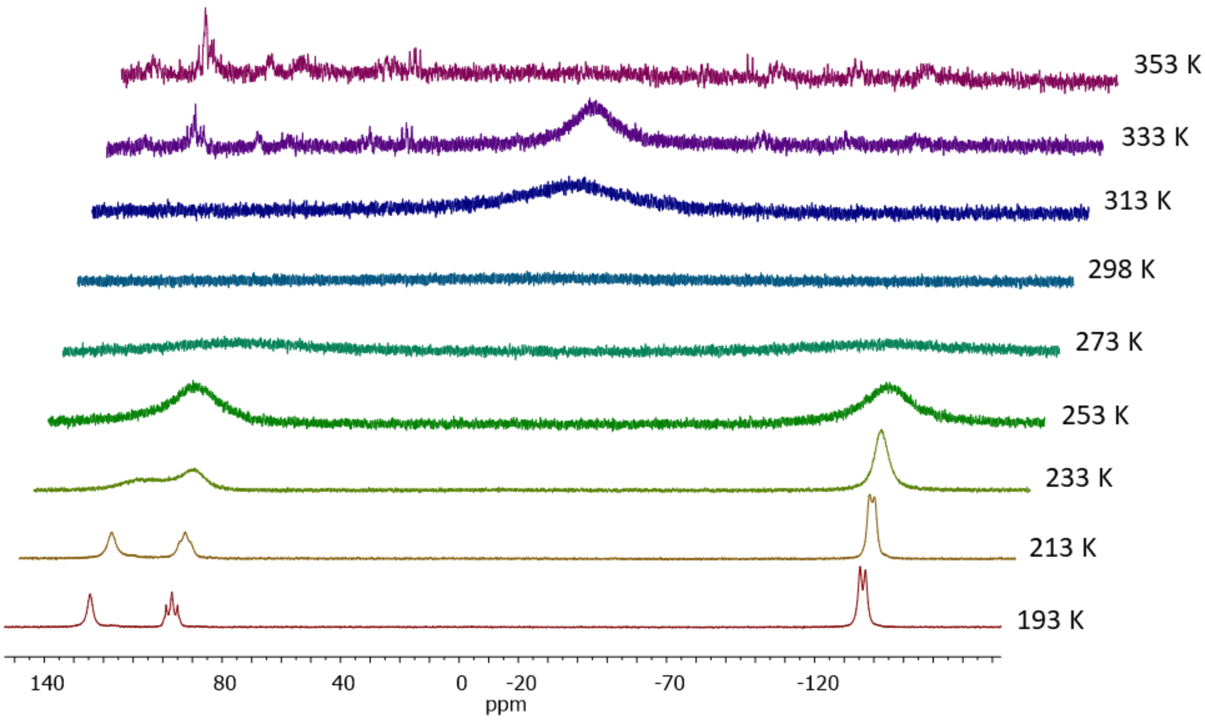

Figure S13. Variable temperature $^{31}P\{^{1}H\}$ NMR (162 MHz, 193-353 K, toluene-d^8) of **4**. Thermal decomposition of **4** starts at 333 K.

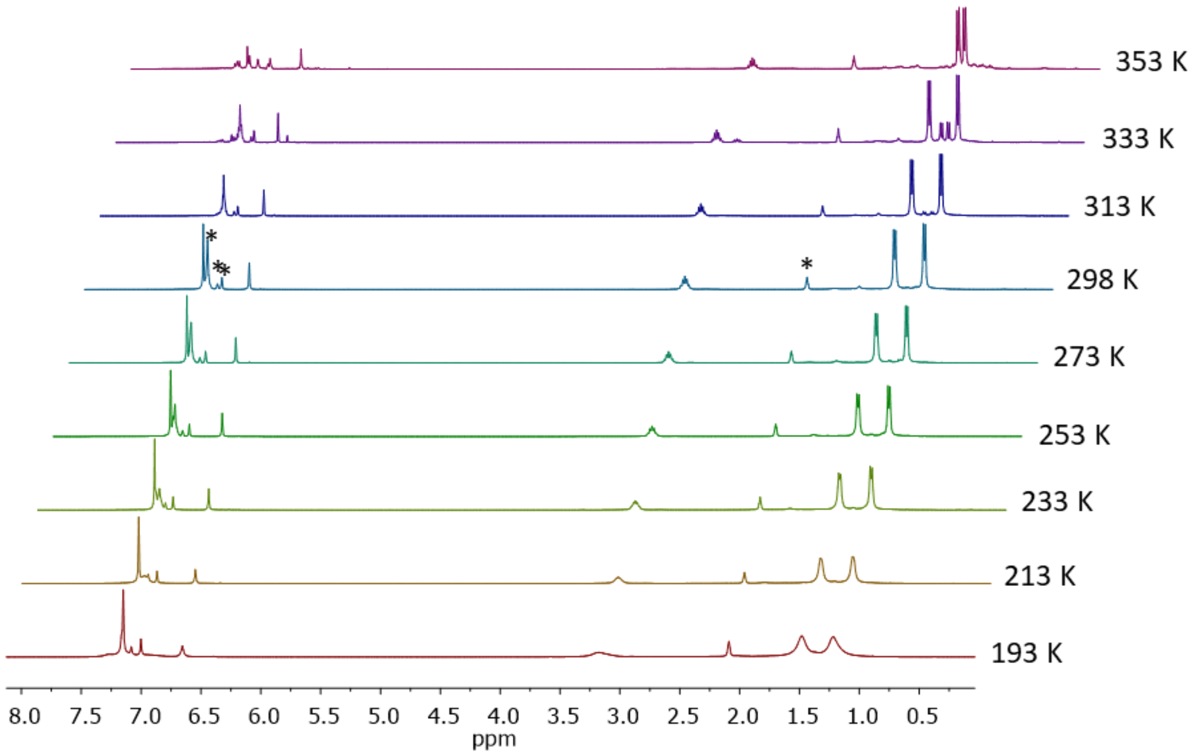

Figure S14. Variable temperature ^{1}H NMR (400 MHz, 193-353 K, toluene-d^8) of **4**. Note that thermal decomposition of **4** starts at 333 K

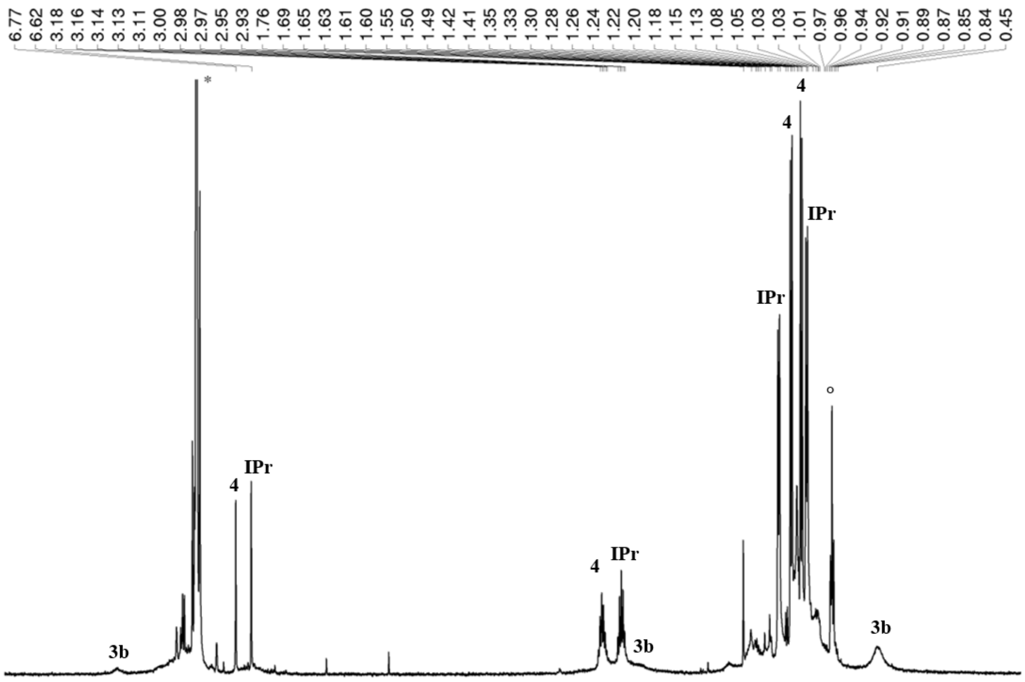

Figure S15. ^{1}H NMR spectrum (400 MHz, 300 K, C_6D_6) of a mixture of free IPr, **4** and **3b** *C_6D_6, °*n*-hexane.

3 UV-Vis Spectra

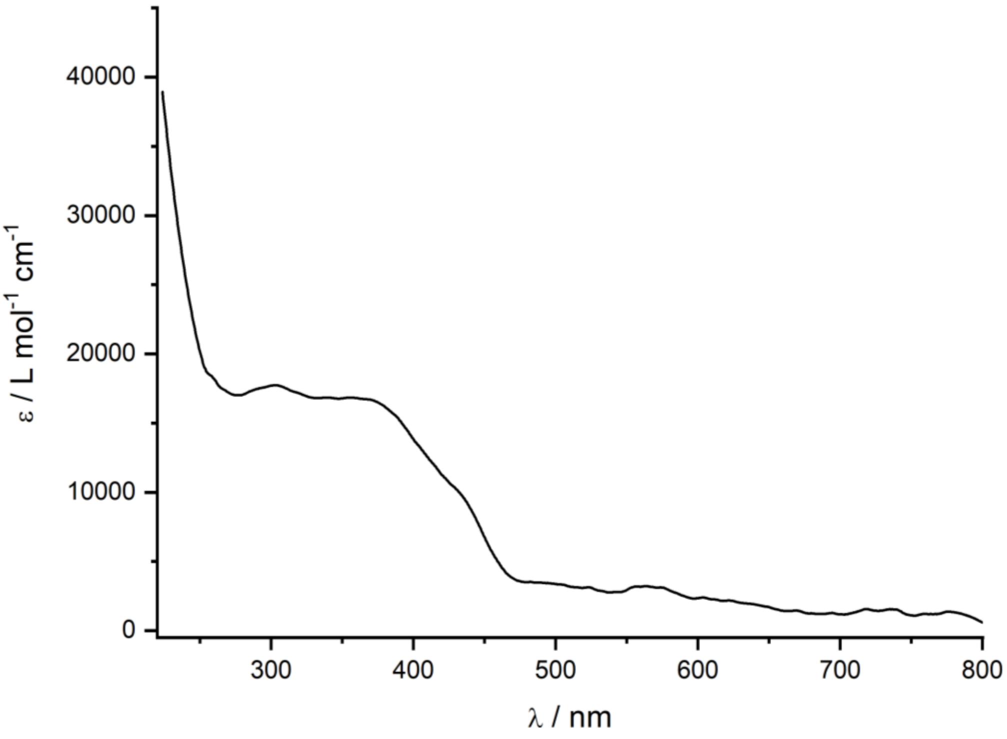

Figure S16. UV/VIS spectrum of **1** recorded in THF.

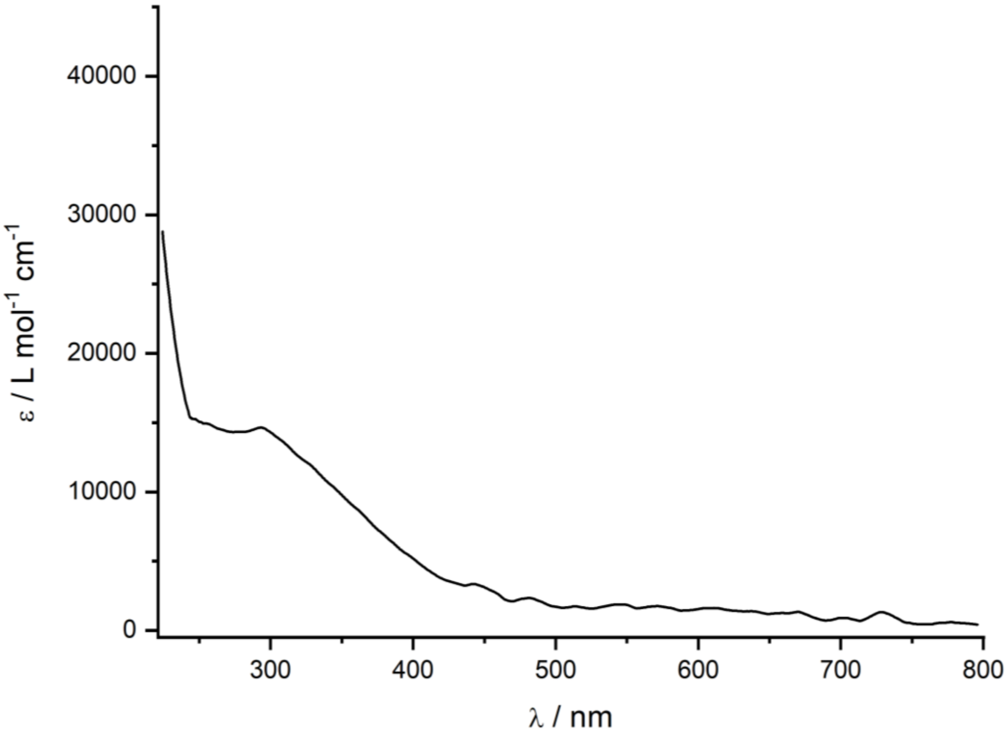

Figure S17. UV/VIS spectrum of **4** recorded in THF.

4 Cyclic Voltammetry

Cyclic voltammetry experiments were performed in a single-compartment cell inside a nitrogen-filled glovebox using a CH Instruments CHI600E potentiostat. The cell was equipped with a platinum disc working electrode (2 mm diameter) polished with 0.05 μm alumina paste, a platinum wire counter electrode and a silver/silver nitrate reference electrode. The supporting electrolyte, tetra-*n*-butylammonium hexafluorophosphate, was dried in vacuo at 110 °C for three days. All redox potentials are reported versus the ferrocene/ferrocenium (Fc/Fc^+) couple. The scan rate is v = 100 mV s^{-1} unless stated otherwise.

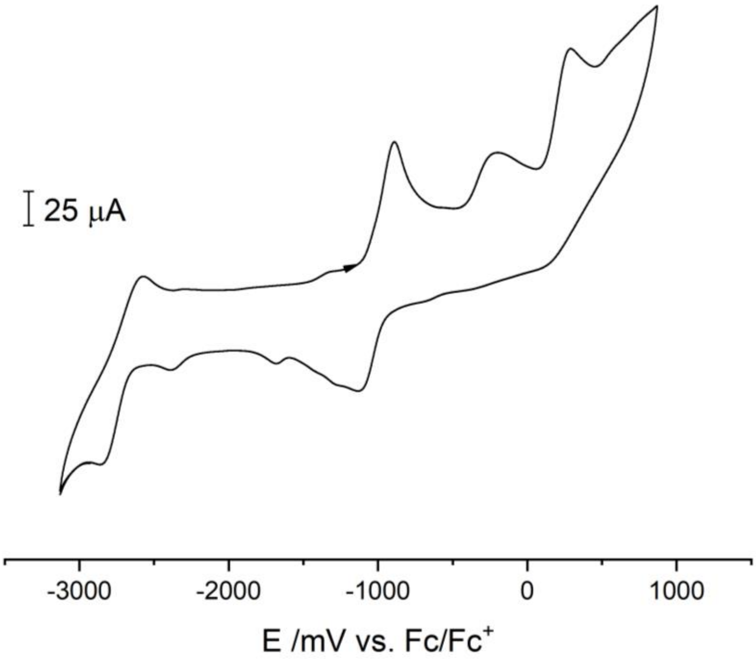

Figure S18. Cyclic voltammogram of **1** recorded in THF/TBAH.

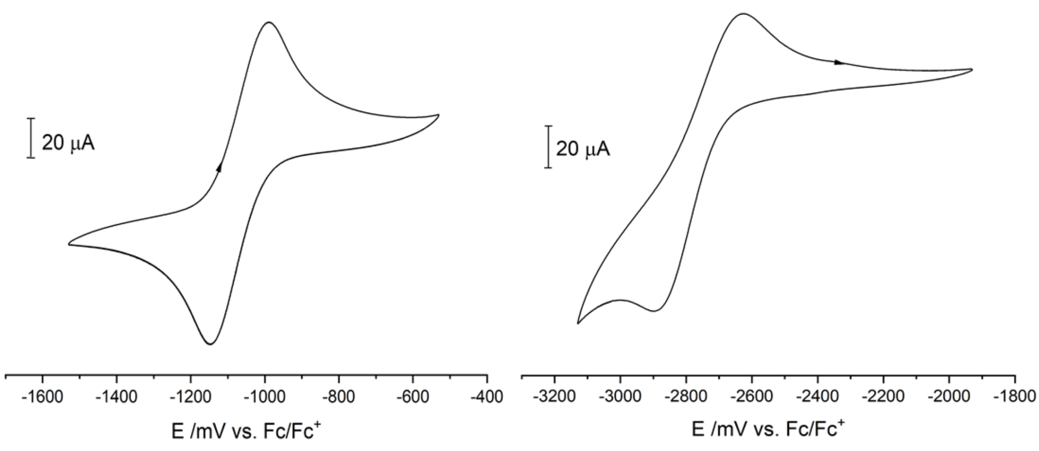

Figure S19. Cyclic voltammograms of **1** recorded in THF/TBAH at two different E ranges.

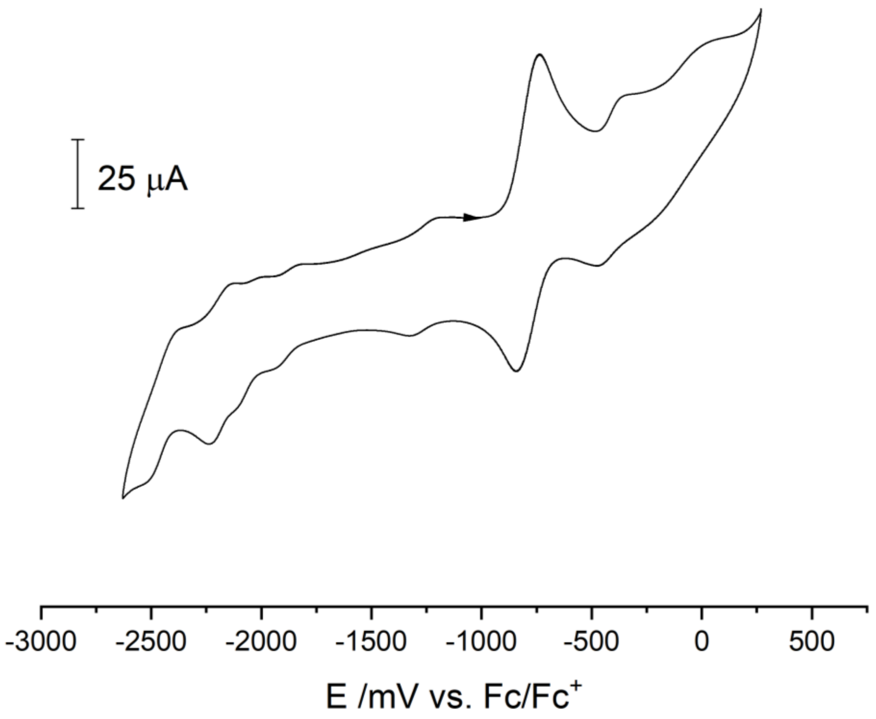

Figure S20. Cyclic voltammogram of **4** recorded in THF/TBAH.

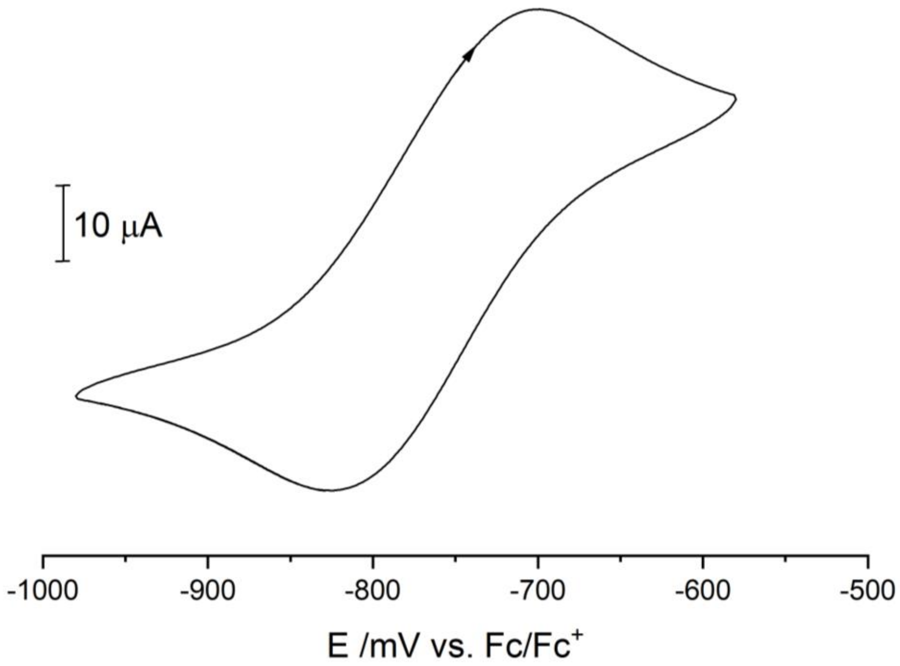

Figure S21. Cyclic voltammogram of **4** recorded in THF/TBAH at a different E range.

5 EPR Spectroscopy

5.1 General Method

Experimental X-band EPR spectra were recorded on a Bruker EMX spectrometer (Bruker BioSpin Rheinstetten) equipped with a Helium temperature control cryostat system (Oxford Instruments). Simulations of the EPR spectra were performed by iteration of the anisotropic g-values, hyperfine coupling interactions and line widths using the EPR simulation program W95EPR developed by Prof. Dr. Frank Neese.

5.2 Experimental and Simulated EPR Spectra

[(IMes)$_2$Ni$_2$P$_5$] (3a)

A crystalline sample containing **2** and **3a** was used for the measurement. This sample was contaminated with another S = ½ system giving rise to the broad resonances. Nevertheless, an axial spectrum displaying hyperfine interaction with a P_5^- ring was observed. Based on the g-values, this spectrum arises from a nickel-centered radical. The sample was measured in toluene glass at 20K using a microwave frequency of 9.650672 GHz, a power of 0.6325 mW and a modulation amplitude 4.000 G. The simulation parameters were: $g_{11} = g_{22}$ = 2.1535, g_{33} = 1.990, W_{11} = 15 MHz, W_{22} = 12 MHz, W_{33} = 4.5 MHz, $A^{31P}{}_{33}$ = 30.0 MHz.

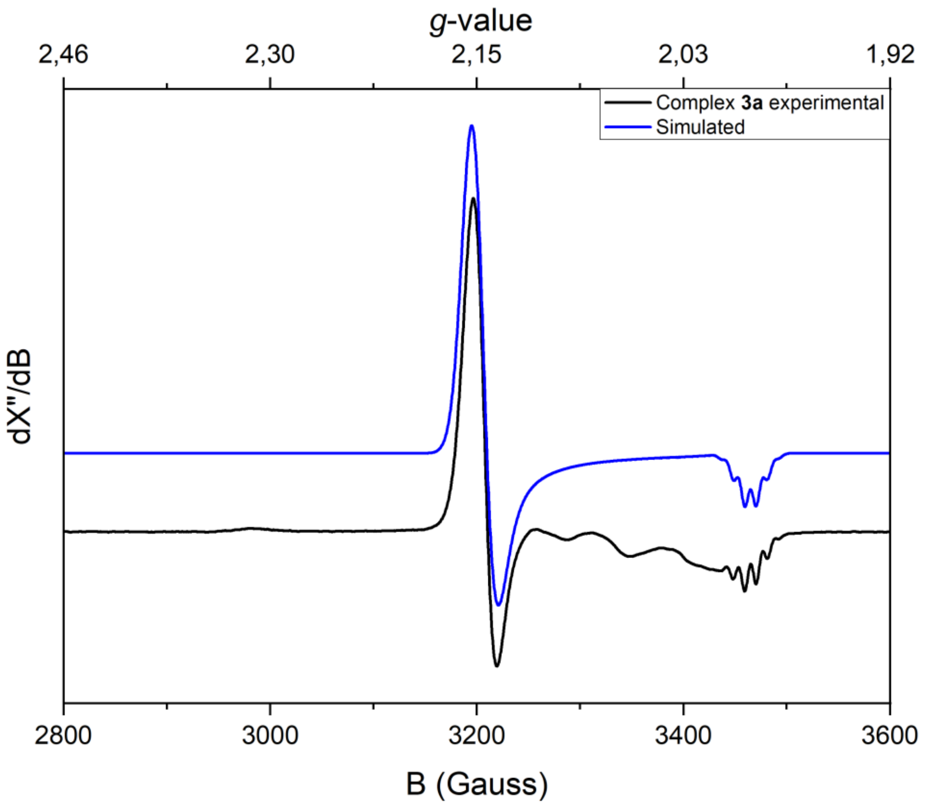

Figure S22. Experimental and simulated X-band EPR spectrum of **3a** contaminated by another paramagnetic species in frozen toluene.

[(IPr)$_2$Ni$_2$P$_5$] (3b) (see main text)

The spectrum was measured in toluene glass at 20K using a microwave frequency of 9.650846 GHz, a power of 0.6325 mW and a modulation amplitude of 4.000 G. The simulation parameters were: g_{11} = g_{22} = 2.186, g_{33} = 1.987, W_{11} = 15 MHz, W_{22} = 13 MHz, W_{33} = 4.5 MHz, $A^{31P}{}_{33}$ = 30.0 MHz.

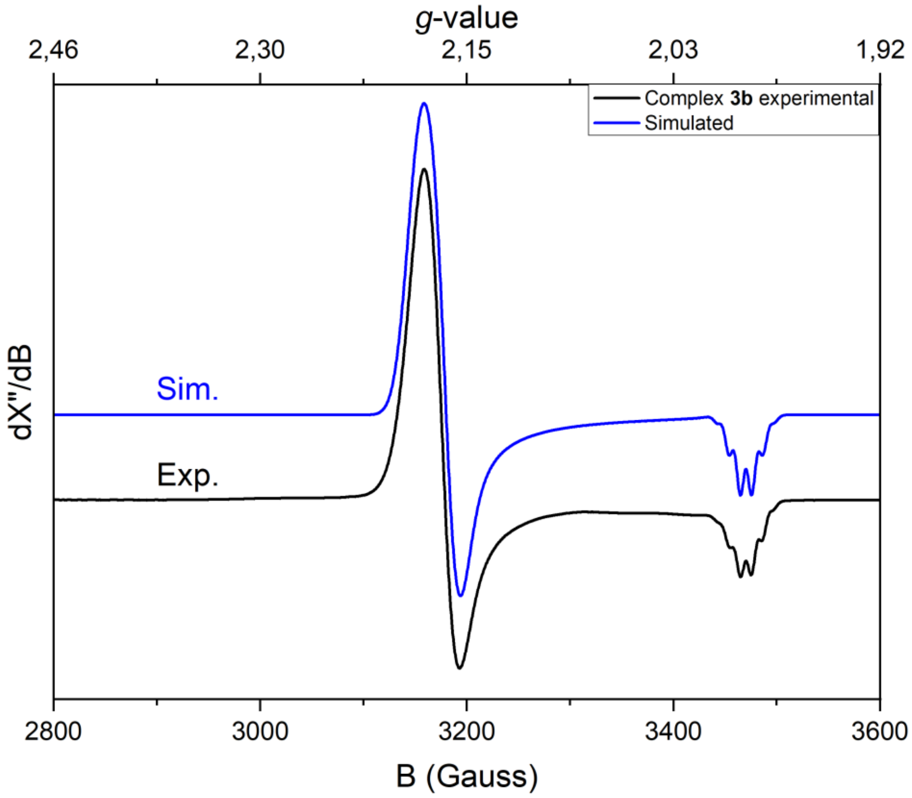

Figure S23. Experimental and simulated X-band EPR spectrum of **3b** in frozen toluene.

6 Single Crystal X-ray Diffraction Data

The single-crystal X-ray diffraction data were recorded on Rigaku Oxford Diffraction SuperNova Atlas or GV1000 Titan[S2] diffractometers with Cu-K_{α} radiation (λ= 1.54184 Å). Crystals were selected under mineral oil, mounted on micromount loops and quench-cooled using an Oxford Cryosystems open flow N_2 cooling device. Either semi-empirical multi-scan absorption corrections[6] or analytical ones[7] were applied to the data. The structures were solved with SHELXT[8] solution program using dual methods and by using Olex2 as the graphical interface.[9] The models were refined with ShelXL[10] using full matrix least squares minimization on F^2.[11] The hydrogen atoms were located in idealized positions and refined isotropically with a riding model.

The crystal of **2** contained several disordered molecules of *n*-hexane which were refined by using the solvent mask command. A solvent mask was calculated and 408.0 electrons were found in a volume of 3086.0 Å^3 in a single void. This is consistent with the presence of one *n*-hexane per formula unit which accounts for 400.0 electrons.

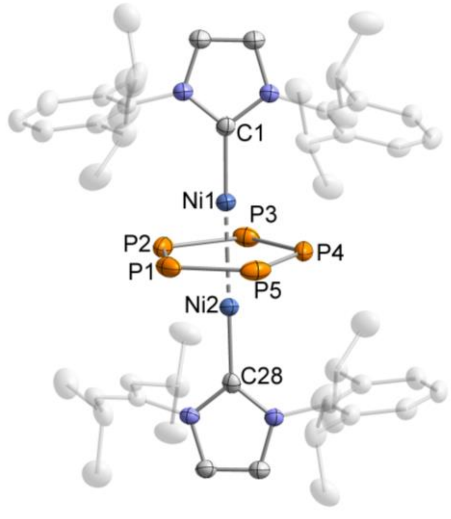

Figure S24. Molecular structure of **3b** in the solid state. Thermal ellipsoids are set at 50% probability level. Hydrogen atoms are omitted for clarity. Selected bond lengths [Å] and angles [°] for **3b**: Ni1−Ni2 2.6813(6), P1−P2 2.1978(11), P2−P3 2.2155(10), P3−P4 2.2160(10), P4−P5 2.2053(11), P5−P1 2.2250(11), P2-P1-P5 107.94(4), P1-P2-P3 107.73(4), P2-P3-P4 108.68(4), P5-P4-P3 107.17(4), P4-P5-P1 108.46(4).

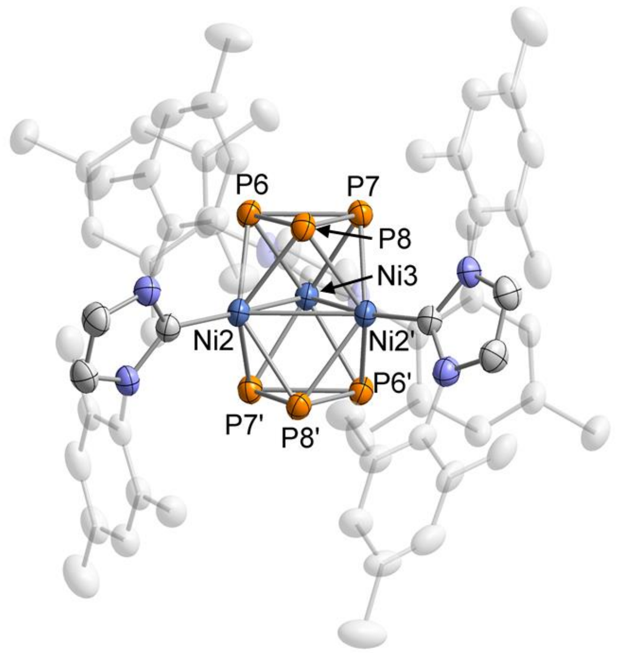

Figure S25. Molecular structure of **2** as part of **2·3a** in the solid state. Thermal ellipsoids are set at 50% probability level. Hydrogen atoms and the molecule of **3a** are omitted for clarity. Selected bond lengths [Å] and angles [°] for **2** (values obtained from the crystal structure of **2** in brackets: Ni2−Ni3 2.4978(10) [2.4834(3), 2.4883(3)], Ni2−Ni2′ 2.6167(12) [2.6432(3)], P7−P8 2.1938(13) [P1−P2 2.2087(5), 2.2049(5)], P7−P6 2.2901(12) [P2−P3 2.2698(5), 2.2822(5)], P6−P8 2.1984(14) [P1−P3 2.2156(5), 2.2116(5)], Ni3-Ni2-Ni2 58.411(18) [57.974(9), 57.793(9)], Ni2-Ni3-Ni2 63.18(3) [64.233(10)], P8-P6-P7 58.48(4) [58.744(16), 58.985(16)] 59.285(16)], P8-P7-P6 58.68(4) [59.030(16), 59.285(16)], P7-P8-P6 62.85(4) [62.226(16), 61.729(16)]

Crystal structures of 4 from *n*-hexane, *n*-heptane, and toluene

The molecular structure of **4** was determined by single crystal X-ray crystallography several times using different solvents for the crystallisations. In each case, a saturated solution of **4** was cooled from ambient temperature to −30 °C. The structure obtained from a crystal grown from *n*-hexane features disorder in the P_8 framework and the IPr ligand. Several samples grown by this method were investigated. All of the data sets feature the same disorder pattern. The structure obtained from a crystal grown from *n*-heptane features disorder in the P_8 framework and is therefore less suitable for interpretation of bond metric data. The structure obtained from a crystal grown from toluene features disorder in the IPr ligand. Bond lengths and angles reported in the manuscript are from this data set. Moreover, the crystal structure of **4** from a crystal grown from Et_2O as solvent was determined and disorder over the P_8 framework was indicated by residual electron density in the cluster core (not included in Figure S26).

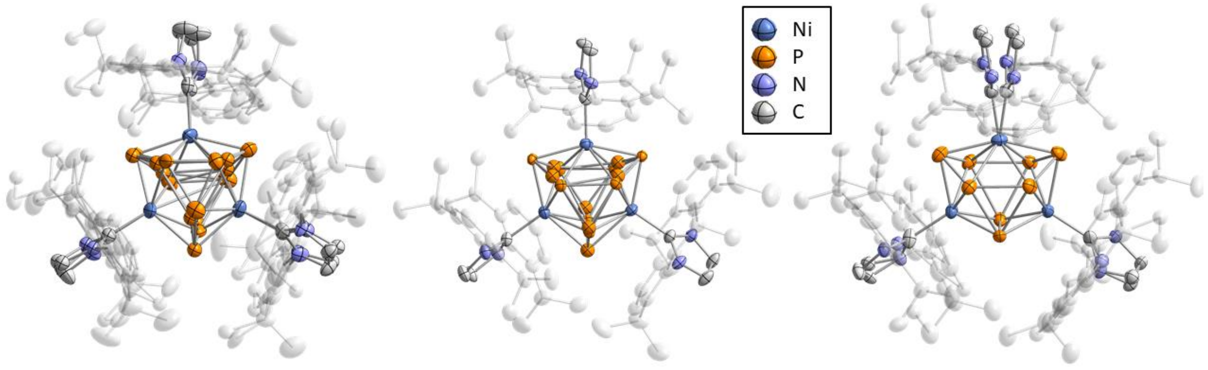

crystal obtained from *n*-hexane R_1 = 9.05% | crystal obtained from *n*-heptane R_1 = 5.12% | crystal obtained from toluene R_1 = 6.77%

Figure S26. Molecular structures of **4** in the solid state depending on the solvent of crystallisation. Thermal ellipsoids are set at 50% probability level. Hydrogen atoms and solvents molecules are omitted for clarity. Depending on the solvent of crystallisation, disorder in the P_8 framework (middle), the IPr ligand (right) or both (left) were observed.

Table S2. Crystallographic data and structure refinement for compounds **1**, **2**, **2·3a**, **3b**, and **4**.

**Compound**	**1**	**2**	**2·3a**	**3b**	**4**
CCDC	1989656	1989653	1989654	1989652	1989655
Empirical formula	$C_{70}H_{80}N_6Ni_3P_4$	$C_{63}H_{72}N_6Ni_3P_6$	$C_{105}H_{120}N_{10}Ni_5P_{11}$	$C_{54}H_{72}N_4Ni_2P_5$	$C_{98.5}H_{128}N_6Ni_3P_8$
Formula weight	1305.41	1275.21	2156.32	1049.42	1819.68
Temperature/K	123.00(10)	123.00(10)	123.00(10)	123.01(10)	89.9(4)
Crystal system	triclinic	monoclinic	tetragonal	triclinic	tetragonal
Space group	P-1	I2/a	$P4_12_12$	P-1	P-4n2
a/Å	11.9677(2)	22.7801(4)	15.54130(10)	10.3749(6)	24.2943(3)
b/Å	16.7171(3)	13.3087(3)	15.54130(10)	14.4469(8)	24.2943(3)
c/Å	17.0685(3)	47.3928(7)	44.4242(3)	19.0051(10)	16.2469(3)
α/°	88.270(2)	90	90	103.126(5)	90
β/°	76.7620(10)	95.1000(10)	90	95.163(5)	90
γ/°	89.6720(10)	90	90	96.316(5)	90
Volume/$Å^3$	3322.55(10)	14311.4(5)	10729.87(16)	2737.8(3)	9589.1(3)
Z	2	8	4	2	4
ρ_{calc}g/cm^3	1.305	1.184	1.335	1.273	1.260
μ/mm^{-1}	2.238	2.477	2.898	2.521	2.307
F(000)	1372.0	5328.0	4500.0	1110.0	3859.0
Crystal size/mm^3	0.289 × 0.268 × 0.194	0.447 × 0.128 × 0.067	0.805 × 0.432 × 0.317	0.208 × 0.169 × 0.052	0.288 × 0.087 × 0.042
Radiation	CuKα (λ = 1.54184)	CuKα (λ = 1.54184)	CuKα (λ = 1.54184)	CuKα (λ = 1.54184)	CuKα (λ = 1.54184)
2Θ range for data collection/°	7.392 to 148.476	6.9 to 147.354	6.942 to 147.918	8.636 to 147.736	7.278 to 148.2
Index ranges	$-14 \le h \le 11$, $-20 \le k \le 20$, $-21 \le l \le 20$	$-28 \le h \le 18$, $-16 \le k \le 16$, $-53 \le l \le 58$	$-19 \le h \le 17$, $-19 \le k \le 18$, $-54 \le l \le 49$	$-9 \le h \le 12$, $-17 \le k \le 17$, $-22 \le l \le 23$	$-30 \le h \le 18$, $-24 \le k \le 30$, $-19 \le l \le 19$
Reflections collected	30320	43060	52935	17586	32746
Independent reflections	13196 [R_{int} = 0.0226, R_{sigma} = 0.0235]	14180 [R_{int} = 0.0260, R_{sigma} = 0.0252]	10776 [R_{int} = 0.0549, R_{sigma} = 0.0313]	10530 [R_{int} = 0.0332, R_{sigma} = 0.0628]	9451 [R_{int} = 0.0522, R_{sigma} = 0.0478]
Data/restraints/parameters	13196/0/820	14180/6/731	10776/0/629	10530/0/602	9451/446/785
Goodness-of-fit on F^2	1.040	1.029	1.106	1.095	1.048
Final R indexes [I>=2σ (I)]	R_1 = 0.0294, wR_2 = 0.0783	R_1 = 0.0286, wR_2 = 0.0750	R_1 = 0.0448, wR_2 = 0.1195	R_1 = 0.0438, wR_2 = 0.1041	R_1 = 0.0677, wR_2 = 0.1655
Final R indexes [all data]	R_1 = 0.0307, wR_2 = 0.0794	R_1 = 0.0317, wR_2 = 0.0774	R_1 = 0.0455, wR_2 = 0.1199	R_1 = 0.0568, wR_2 = 0.1180	R_1 = 0.0791, wR_2 = 0.1730
Largest diff. peak/hole / e Å^{-3}	0.30/-0.40	0.31/-0.24	0.98/-0.28	0.79/-0.45	0.82/-0.38
Flack parameter			0.009(10)		0.016(13)

7 Quantum Chemical Calculations

General Methods

All calculations were performed with the ORCA program package.[12] All calculations were conducted in the gas phase. The RI approximation was used for GGA calculations,[13] whereas the RIJCOSX approximation was used for hybrid-DFT calculations.[14] Geometry optimisations have been carried out at the BP86-D3BJ/def2-TZVP level of theory.[15] Thereby, the aryl substituents at the NHC moieties were truncated to phenyl rings [NHC = 1,3-diphenylimidazolin-2-ylidene (IPh)]. The transition state for the fluxional process of **1** in solution was located using relaxed surface scans followed by a saddle-point optimisation.

Intrinsic bond orbitals (IBOs) have been constructed from the occupied BP86 orbitals according to Knizia *et al*.[16] To estimate the electron count within the clusters, the composition and shape of the respective IBOs was analysed. Thereby, an IBO with a Ni contribution greater than 65% are identified as occupied 3d orbitals. This criterion ensures that only significant bonding interactions between the cluster atoms are taken into account when determining the number of cluster electrons. Orbitals with a comparably low Ni contribution (65% to 80%) may indicate back-bonding from Ni in P-based orbitals, offering additional stabilisation.

Orbital Pictures and Compositions

[$(IPh)_3Ni_3P_4$] (**1′**):

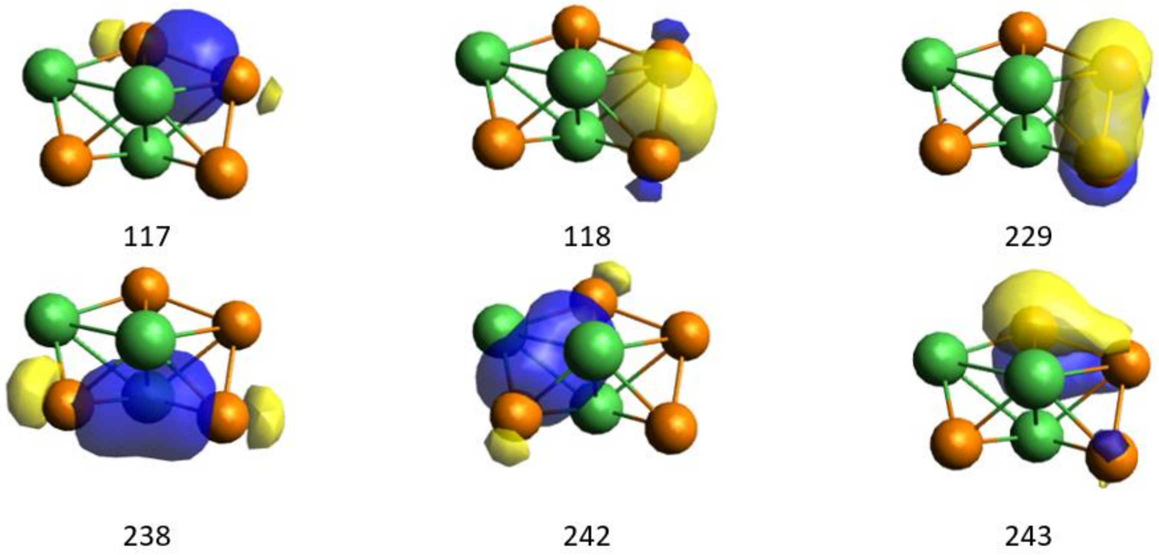

Figure S25. Intrinsic Bond orbitals of **1′** showing significant bonding interactions between the cluster atoms. Surface isovalue = 0.06. NHC groups at the Ni atoms have been omitted for clarity.

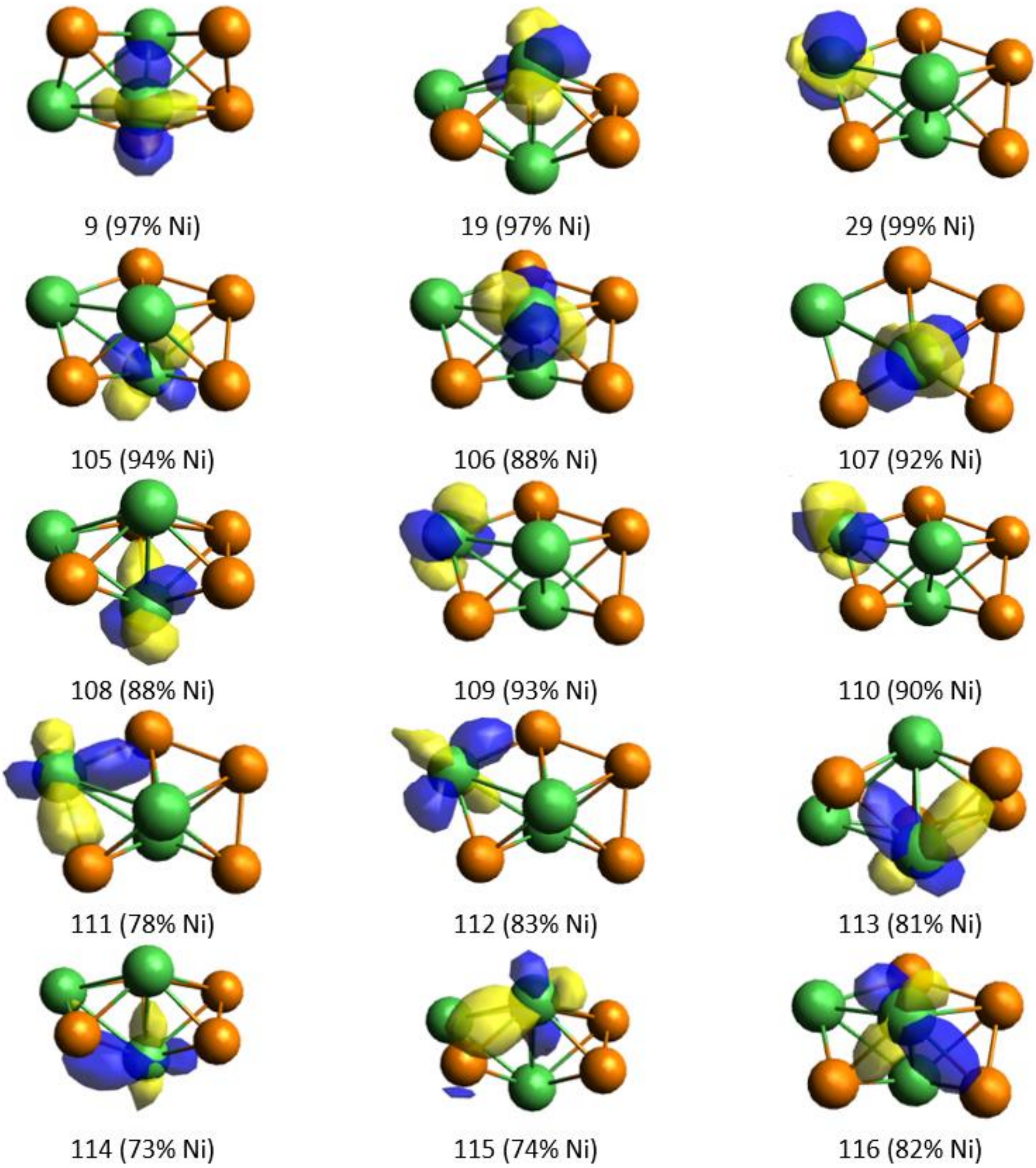

Figure S26. Intrinsic bond orbitals of **1′** showing the filled 3d-orbitals at the Ni atoms (highest contribution of a single Ni atom is given in parentheses). Surface isovalue = 0.06. NHC groups at the Ni atoms have been omitted for clarity.

[$(IPh)_3Ni_3P_6$] (**2′**):

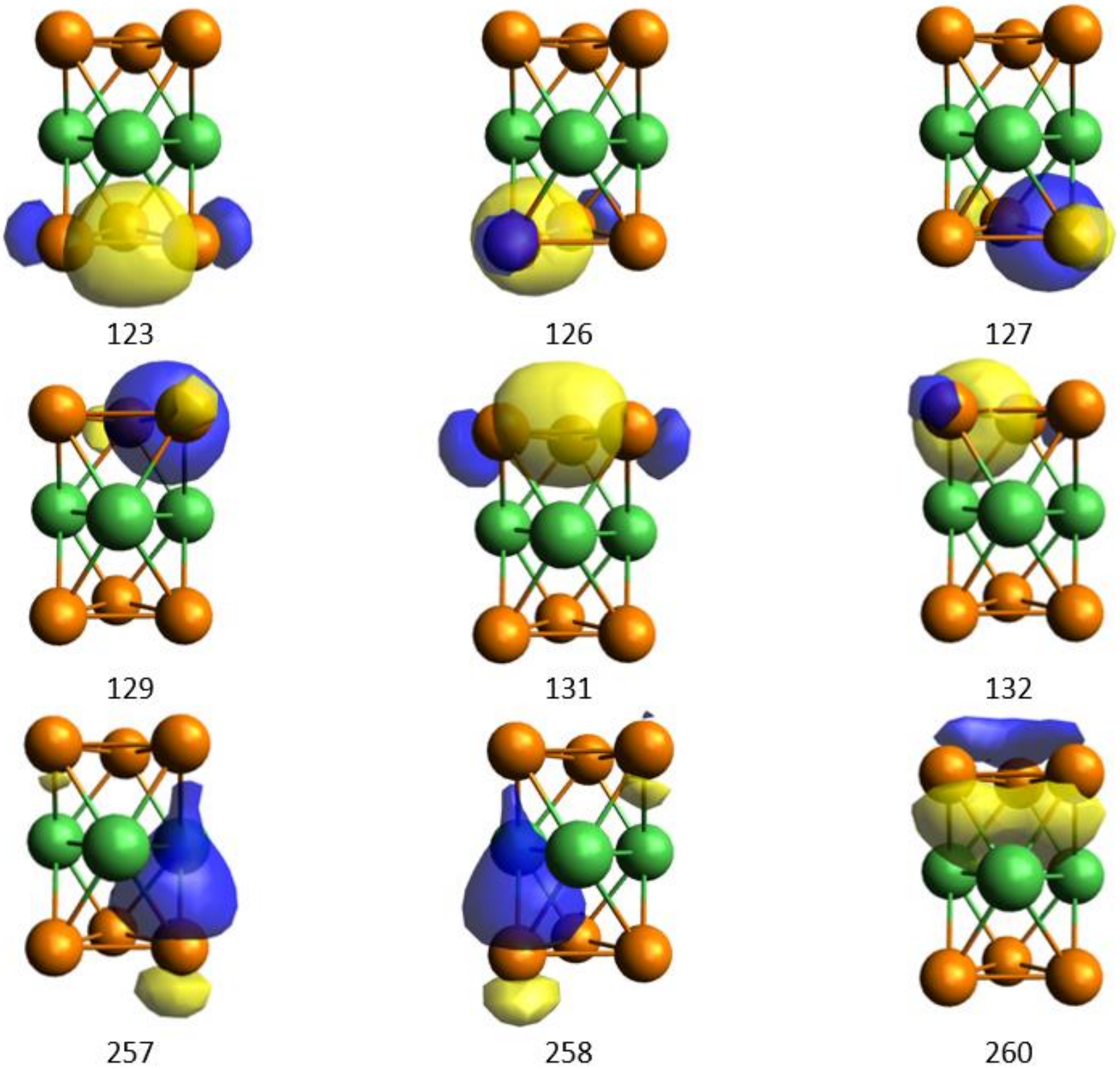

Figure S27. Intrinsic Bond orbitals of **2′** showing significant bonding interactions between the cluster atoms. Surface isovalue = 0.06. NHC groups at the Ni atoms have been omitted for clarity.

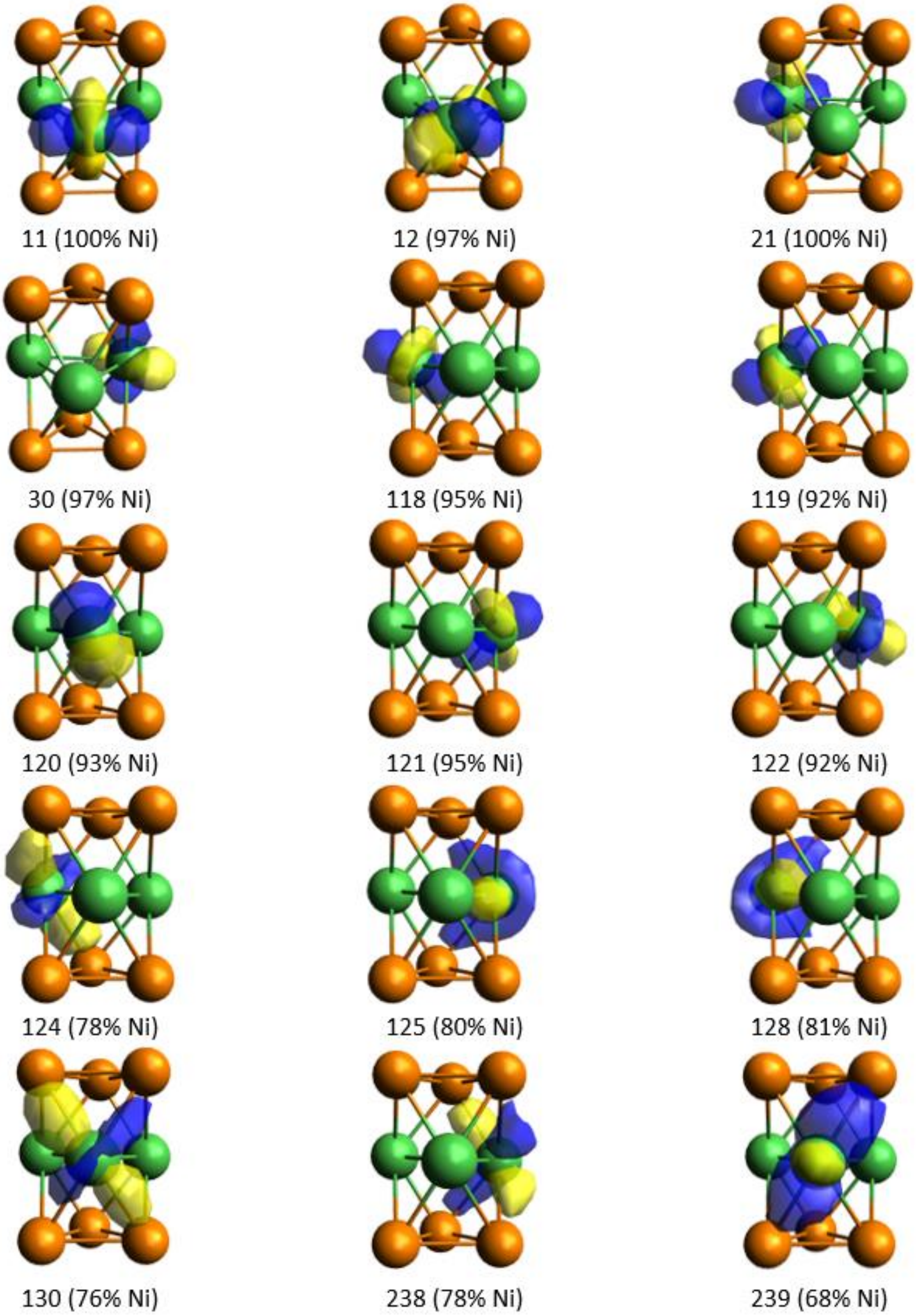

Figure S28. Intrinsic bond orbitals of **2′** showing the filled 3d-orbitals at the Ni atoms (highest contribution of a single Ni atom is given in parentheses). Surface isovalue = 0.06. NHC groups at the Ni atoms have been omitted for clarity.

[(IPh)$_3$Ni$_3$P$_8$] (**4′**):

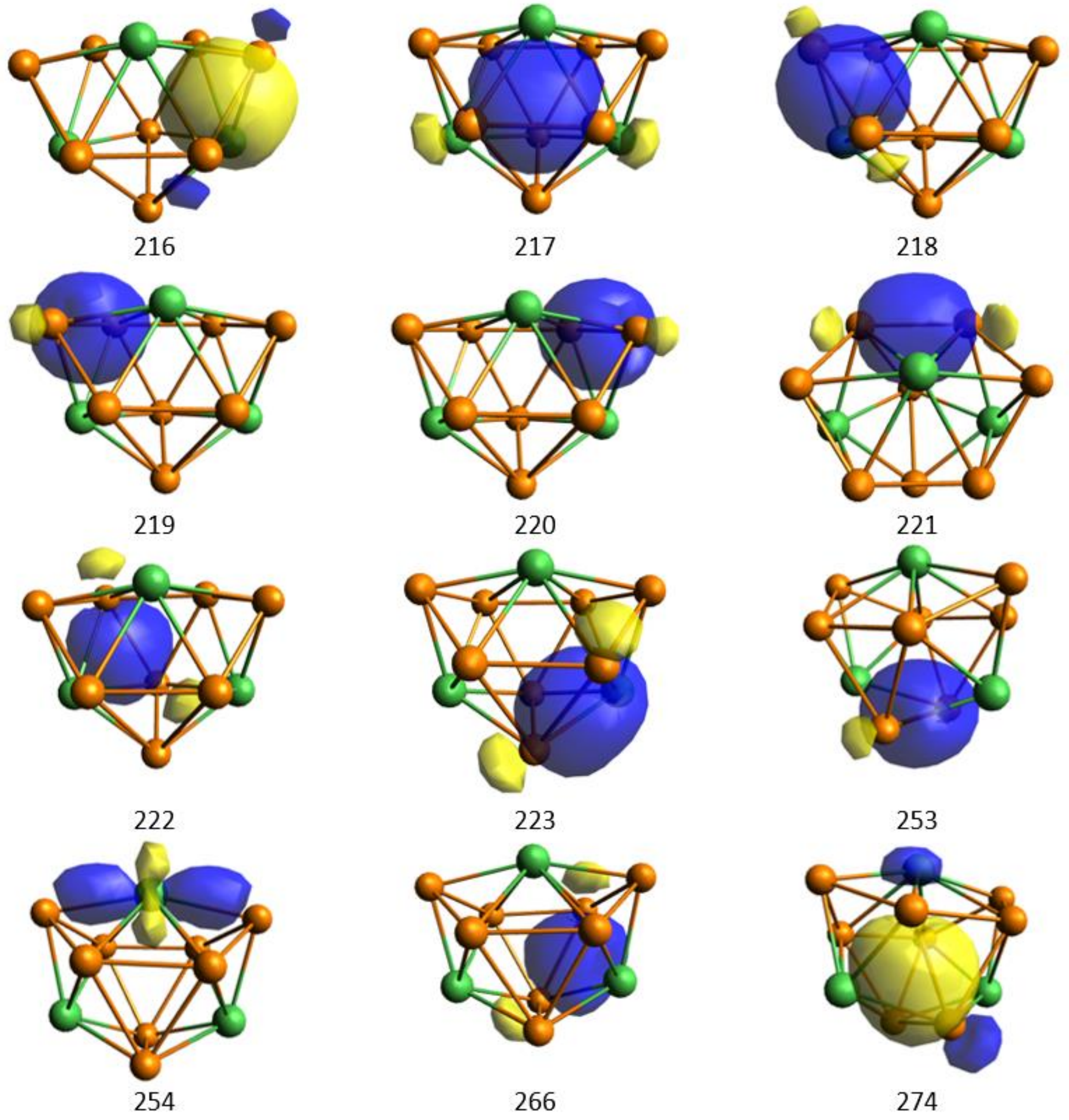

Figure S29. Intrinsic Bond orbitals of **4′** showing significant bonding interactions between the cluster atoms. Surface isovalue = 0.06. NHC groups at the Ni atoms have been omitted for clarity.

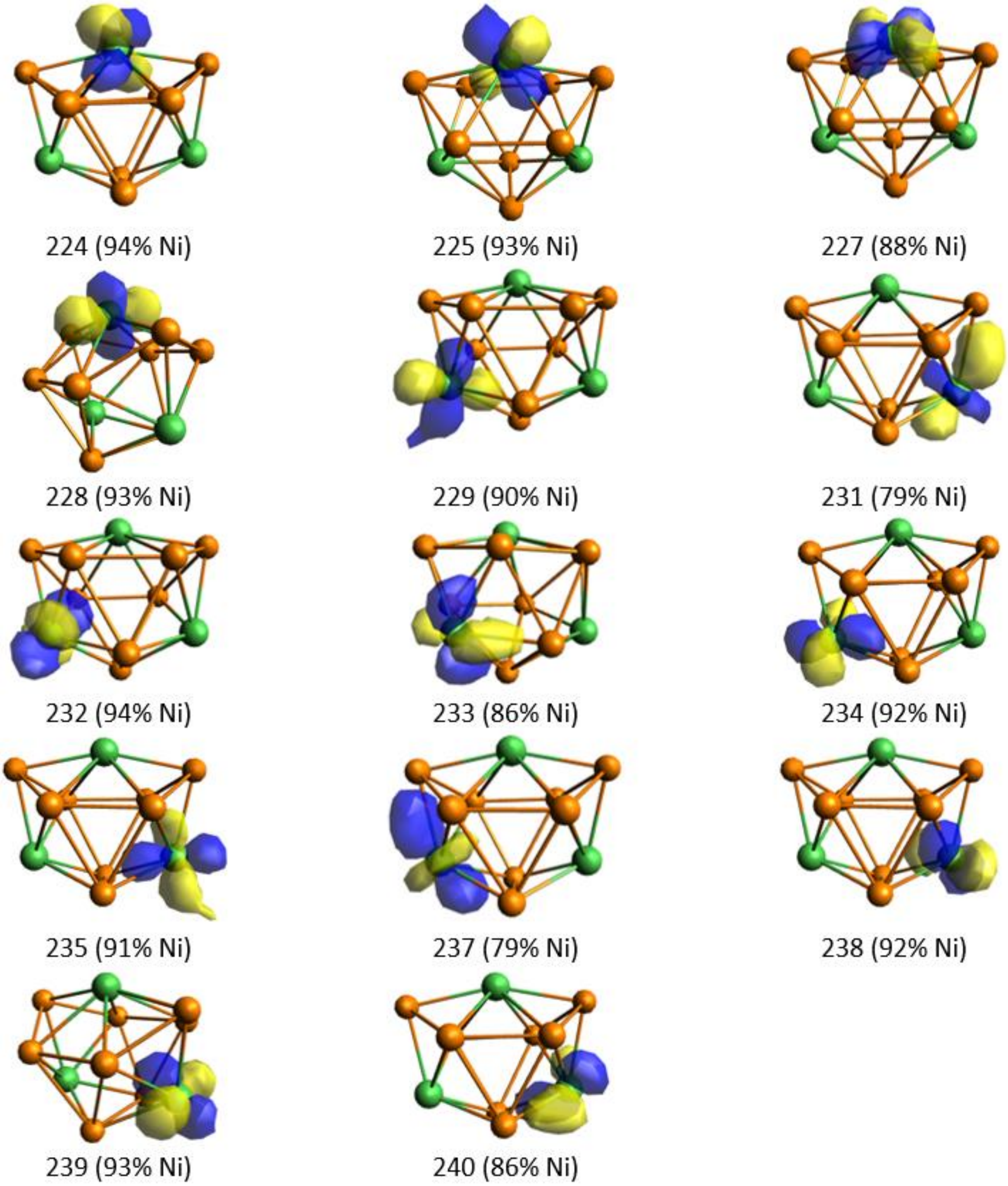

Figure S30. Intrinsic bond orbitals of **4′** showing the filled 3d-orbitals at the Ni atoms (highest contribution of a single Ni atom is given in parentheses). Surface isovalue = 0.06. NHC groups at the Ni atoms have been omitted for clarity.

Fluxional behaviour of 1 in solution

DFT calculations at the BP86/def2-TZVP level of theory gave more insights into the fluxional behaviour of **1** in solution. The presence of only two major signals in $^{31}P\{^{1}H\}$ NMR can be attributed to either an exchange process between the phosphorus atoms or the formation of a symmetrical isomer. Starting from the asymmetrical isomer [(IPh)$_3$Ni$_3$P$_4$] (**1′**), the symmetrical isomer **1*** can form (Figure S27, left side). This symmetrical isomer is slightly (0.3 kcal/mol) more stable in energy compared to **1′**. Moreover, **1′** can undergo a change in connectivity via a transition state that is 2.6 kcal/mol higher in energy than **1′**.

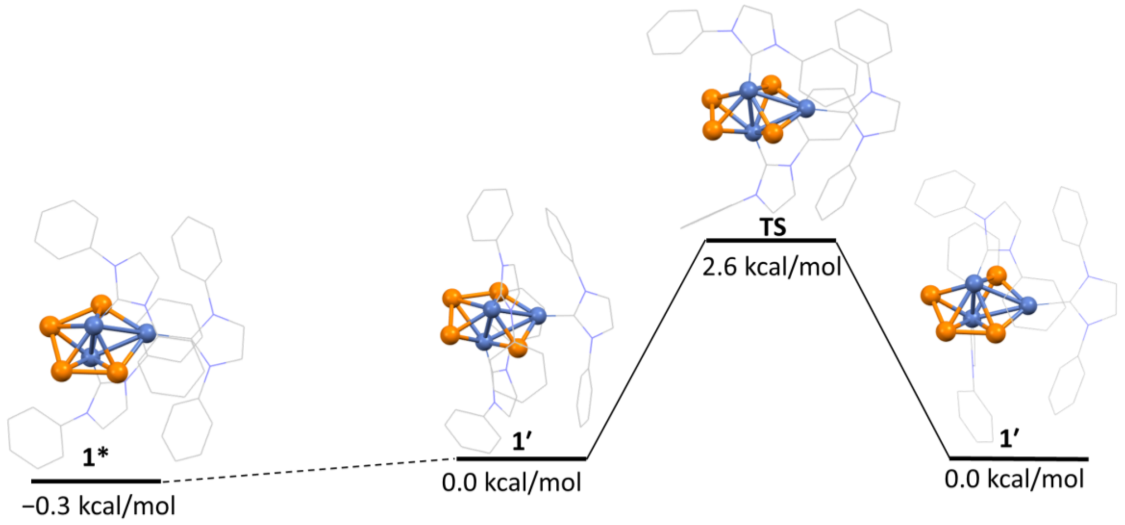

Figure S27. Isomerisation of the asymmetrical isomer **1′** to the symmetrical isomer **1*** (left) and connectivity change in **1′** (right). Note that solid lines are transformations with a found transition state, and the dashed line indicates a transformations for which no transition state was found.

Cartesian Coordinates of Optimised Structures

[(IPh)$_3$Ni$_3$P$_4$] (**1′**, unsymmetrical isomer resembling crystal structure)

Ni	13.40254838552172	14.49878132866253	13.25542264117263
Ni	13.27923858827665	12.42766449516554	12.03247448859933
Ni	10.99031961685278	13.33797674984631	13.1609828707068
P	12.62425224740829	12.57151180937054	14.31255211168585
P	12.10851354131078	14.15530912200754	11.5522723942295
P	14.78696274259674	12.51453407106275	13.96863788990859
P	15.14983331403212	13.63922075651268	12.13535334001406
N	8.55098912436172	12.47075086961428	14.46744784423685
N	8.24985760154552	12.46542992046274	12.32526347038900
N	12.53708551998850	11.26947275802396	9.43655486919104
N	13.45159392484180	16.95771795873294	14.93756879233807
N	11.71968584992447	10.14565406508542	11.08921873459813
N	13.17672659925197	17.36774785407717	12.83379223048311
C	8.05590590561257	11.55646666501145	10.06847411148383
C	8.45548118665200	12.58872398795793	10.92473608613295
C	9.17746021428511	12.83187570424171	13.28714933047962

C	10.70218169778488	8.96264923565387	14.99411577153180
C	12.97802752801767	17.20663947517704	11.42862210131686
C	7.12510398231665	11.86658415480065	12.89522200815618
H	6.27450681177024	11.55392849502686	12.30331981182616
C	9.06302446685090	13.73809541891843	10.40974186435422
C	12.93582638726670	16.78934449718794	17.31734596837483
C	7.31821996747825	11.86866160289894	14.23998721143582
H	6.67042523295314	11.55079652335151	15.04689780437356
C	14.00573645830513	16.68435093304871	10.64056316709328
C	11.73717938121495	17.52715223775747	10.87454336514642
C	13.40029970916043	16.32187950839247	13.70730127413558
C	10.03627878690448	9.59299623220840	12.75616802337356
C	13.26484815941090	12.20138160014134	8.64382953060401
C	14.70140400739550	13.93306612343024	6.99636315241958
C	9.69888215554111	9.20598057571736	14.05274146322677
H	8.64719958884950	9.12087209134370	14.32824464243476
C	12.60014373056699	11.17284765631316	10.81328486738966
C	9.16101312089273	12.62592453372233	15.74411426805077
C	12.61764720873132	12.85548112004611	7.59230650417697
C	8.26461235906504	11.67868213604687	8.69381521344570
H	7.95401685080917	10.87162245476669	8.02934651731264
C	12.39103350490898	9.46604155092139	13.33221963313015
C	14.62242374973163	12.42264890351818	8.89238257819330
C	9.91142247660450	13.77619532753831	16.00925926743267
C	13.34126346632135	13.71567559414477	6.76434284522927
H	12.83684992453742	14.22131912499493	5.93987600016023
C	9.29061147387334	13.84048014394541	9.03867888728912
H	9.78956574543822	14.72880261786077	8.65070504661985
C	14.41375891214246	15.16529847696743	16.28366052834721
C	12.04404812460123	9.09345326788833	14.63119198625696
H	12.83067423688958	8.92139384885464	15.36565541253513
C	11.62863708163822	10.35997029524156	8.89566982158951
H	11.47035412766304	10.27262912886893	7.82927710142772
C	9.05413804401602	11.61171654710858	16.69815391376336
C	14.53218458913133	14.50631242413233	17.50666066775784
H	15.14805196601202	13.60847142790965	17.56321490985498
C	13.77156612997106	16.44922476618025	9.28778519832848
H	14.55311052452792	16.00124109777468	8.67689404236948
C	13.60444402909129	16.29977033298099	16.18788208495650
C	8.88773007704812	12.81655247563876	8.17601374272588
C	11.51634150357274	17.30141525935600	9.51372966836268
H	10.54407759196476	17.53680704455358	9.07891754196589
C	15.33370409468058	13.29267165238888	8.06753468548808
H	16.39268451806390	13.46437205291303	8.26287320159884
C	11.11555582778616	9.65477501513741	9.93432883818988
H	10.41748587291342	8.82852478374045	9.95842409244030
C	11.38155213072236	9.71519436628365	12.40180722935320
C	12.52720554291136	16.74712306273459	8.72557159695482
C	13.87838570726415	14.99231664425644	18.64121172095514
C	10.57787118162849	13.89430787225441	17.22638040123270
H	11.19033405211202	14.77387175263972	17.41799365739264
C	13.08834749530943	16.14190534848015	18.54480955947718
H	12.56442968451550	16.52491371108792	19.42138819466606
C	9.71596675890646	11.74475109672382	17.92088997012486
H	9.64457216828862	10.94613977025692	18.66032425258349
C	13.24821077427716	18.33394849707302	14.81445049128096
H	13.30473229360054	19.00414220820696	15.66173946138918
C	13.07066124233057	18.58782989244656	13.49359348681423
H	12.92189676625412	19.51733507916859	12.95912434237504
C	10.48722619255119	12.87995175199121	18.18199930449617
H	8.49156895358548	10.70712103647646	16.46865234539106
H	11.02335264542193	12.97340274654082	19.12705585018604
H	9.99332058019833	14.55038507176491	15.24551472190706
H	7.59870486797370	10.65485305176035	10.47791002129475
H	9.35895893650163	14.53369214176118	11.08915503320874
H	9.05262479928678	12.90628538145203	7.10130274015629
H	15.26694995486820	14.60376654685408	6.34804017088388
H	12.34752074003028	16.53908797277592	7.67042736660657
H	14.94318690731779	14.80390677137685	15.40385911416805
H	14.95481917995649	16.42059141445787	11.10444530576373

H	10.94273119895807	17.90496423361543	11.51870797788532
H	10.43937076424185	8.68162428290480	16.01414966190593
H	12.27633673126722	17.65329355223411	17.23222725184360
H	13.98208650772995	14.47825010953237	19.59733936534850
H	13.43225669126073	9.58708923653894	13.03947027957496
H	11.54920657923699	12.70150811448795	7.44414700988496
H	15.10210344848175	11.91621100394475	9.72689707315775
H	9.26495897613322	9.83633839557234	12.02643276463803

[(IPh)$_3$Ni$_3$P$_4$] (**1***, symmetrical isomer)

Ni	13.52061533126539	14.69864224947701	13.31043067031789
Ni	13.61127426906433	12.26600149531655	12.16150826674840
Ni	11.31531535282302	13.67871931629193	12.22541603097969
P	12.47577745098996	12.87783647373341	13.99845085297506
P	13.24501585654460	14.23249121624232	11.15085268978842
P	14.79736783269966	12.82470760394243	14.09972681979870
P	15.33964343808701	13.81246578514062	12.21112975326833
N	8.62083763678387	13.19208075647942	13.44621535310505
N	8.64016586245040	12.87665932955404	11.30495676757638
N	12.96629361234433	10.82744644702528	9.71894787476033
N	13.41694663245016	16.61177332645234	15.49838640518565
N	11.65888527074766	10.18720270301035	11.31728684074294
N	11.88725290085424	17.08668089263850	14.05005667110248
C	8.51218364477837	11.71207455718691	9.16074929093443
C	9.07961616437330	12.70564724692854	9.96694477836620
C	9.47566609467056	13.24478556897576	12.35437114114985
C	9.80145985573038	9.61340809776977	15.05087220138551
C	11.03087210610926	17.06314443344253	12.91171243805254
C	7.34548834149371	12.60342980018800	11.73895623830557
H	6.54226240397489	12.35414681895355	11.05802025062951
C	10.09534935097310	13.52454150131745	9.46435514972894
C	14.34997326837318	15.49269796572877	17.45475357860399
C	7.33510512255036	12.80117437513310	13.08097458482758
H	6.52066336229177	12.75362603124098	13.79176658485812
C	11.57987230549724	17.00193030025212	11.62911024341733
C	9.64328284670112	17.07090928477849	13.09200048024158
C	12.95546434074818	16.22653863902821	14.25520665805649
C	9.64414650762471	10.00910930961245	12.66927632027266
C	14.01172629159339	11.44856467776305	8.98082284975779
C	16.04605158816923	12.71064827426479	7.55127616981357
C	9.02798456667962	9.81230922121785	13.90477372695340
H	7.93944976785808	9.83391513635713	13.96812970846885
C	12.75408679117958	10.99604510493433	11.07657698806461
C	8.99683271534875	13.44969833488654	14.79317312415468
C	13.75532633466892	11.94039618353710	7.69835998516146
C	8.96120711234766	11.54588183958519	7.84929194609056
H	8.51762434589152	10.76973578777235	7.22470756348592
C	11.81905160918491	9.76985007557184	13.71844153578199
C	15.28553425376572	11.56202655950039	9.54362797450221
C	9.97108705602109	14.41059705734697	15.07004431869510
C	14.77669414408144	12.56975971806583	6.98498898050376
H	14.57293342907334	12.96480839573297	5.98887888327521
C	10.55695402725793	13.33359786369756	8.16413559846749
H	11.36374859334538	13.96639328049718	7.79330277990080
C	15.71685478568329	15.80983066896182	15.47086982370303
C	11.19440084118022	9.58976493536966	14.95231337130610
H	11.80505638144215	9.43841729081161	15.84243646358641
C	12.01355541801755	9.98790796795552	9.14628444694293
H	12.03336613391946	9.72387456660028	8.09781446440363
C	8.39953180353340	12.73233174033038	15.83523885767407
C	16.76988431823411	15.14620554907319	16.09864618655920
H	17.70451187280695	14.99576292590434	15.55863341211271
C	10.73162465009910	16.93768638249723	10.52310542973911
H	11.16526876635450	16.88150424207597	9.52433945396270
C	14.51127049608698	15.97523385632171	16.15522799628209
C	9.98787418722400	12.35091072018350	7.34898945311132
C	8.80359655661745	16.99470909857128	11.97979349914119

H	7.72280479439154	16.97633384494228	12.12310147149303
C	16.29629453650125	12.20134915937508	8.82865433813891
H	17.28226223109446	12.30818250430127	9.28088891649575
C	11.19382028879025	9.58678531057590	10.14812941921291
H	10.35961203805710	8.89836796785324	10.15028245569428
C	11.03810722890535	9.99096769347137	12.58147015541385
C	9.34503369318538	16.92409916051269	10.69360320739960
C	16.62318543528581	14.66450224682426	17.40282048554949
C	10.34932484926140	14.65394030321994	16.38904813258647
H	11.11789595384138	15.40001898447942	16.58904805567535
C	15.41576806939917	14.84186032918453	18.08089495599380
H	15.29147690440668	14.45411181227418	19.09251123602598
C	8.77869216643054	12.98633982354409	17.15401866452860
H	8.31822442576855	12.41809815904777	17.96303510309938
C	12.65231521103761	17.63506775203704	16.05062560074263
H	12.88836679460357	18.08036980678528	17.00882430293454
C	11.68873830775642	17.93375682197464	15.14013133158070
H	10.91880314853274	18.69436000208638	15.14441790325885
C	9.75545986276697	13.94593873613447	17.43558353056266
H	7.66868543789514	11.95497110300577	15.61612819758658
H	10.05556901877532	14.13360455715569	18.46717043103285
H	10.43690036388949	14.93368910334155	14.23865930782608
H	7.73954543951169	11.05536525561733	9.56203965444372
H	10.51150187420283	14.31527473718335	10.09099784345457
H	10.33909818687201	12.21408748249823	6.32514187547517
H	16.83874615817929	13.21576503887814	6.99835455414503
H	8.68809246445077	16.85167542460916	9.82635445938518
H	15.80629174060738	16.17331472182697	14.44861214708520
H	12.66117605074960	16.99796998752161	11.51054656342339
H	9.22799396262120	17.08795904342691	14.09967753167848
H	9.32048910290294	9.47922007571172	16.01999383175490
H	13.38771070436928	15.59931087714254	17.95665160360177
H	17.44753805968634	14.13905896309555	17.88594638116660
H	12.90340414041574	9.76210298618714	13.62828189192635
H	12.75241138251659	11.85950814699348	7.28179675729658
H	15.46543641456535	11.16907454578834	10.54339066164447
H	9.04953755711257	10.20499855016434	11.77758711390496

[(IPh)$_2$Ni$_2$P$_5$] (**3′**)

Ni	6.35153355909917	3.51035264259057	3.92821775530809
Ni	7.26827636922801	1.57596381903156	5.49824776115351
P	6.29604726150640	1.23333799145857	3.40146764796528
P	5.02783979569714	1.99453314657066	5.08184432802988
P	6.22225552853812	3.50799171206316	6.25405555570411
P	8.27217218984466	2.27840374933312	3.52833189412974
P	8.22605028279849	3.68976028381575	5.28794720484267
N	9.22374260700717	0.35151347566716	7.32616733163511
N	4.79066258302546	4.65564104339115	1.70912713141137
N	4.90424313584551	6.01328990290763	3.37767702026691
N	8.21822530888984	-1.13805785604697	6.13848072473882
C	9.91063242700632	-0.84462143904014	7.52478348300187
H	10.74679764119922	-0.92336035894489	8.20803776514746
C	3.95652413151919	6.53926605089410	2.50171966840692
H	3.46248568874924	7.48480170912806	2.68635389010003
C	9.27565940444046	-1.78524082166664	6.77529512970270
H	9.44644100687463	-2.84948169866664	6.67348695144428
C	7.31396998289450	-1.75318351696913	5.22464805524593
C	8.17737723753667	0.20548728963900	6.44138428220471
C	3.88493708763850	5.68221011536585	1.44816516903127
H	3.31651187875225	5.72985324908517	0.52794900298524
C	5.42265016004854	4.82021276572375	2.92263742721896
C	9.58227310061523	1.60658276896107	7.89905695131228
C	5.00619761299893	3.52277903695160	0.87162376250794
C	5.93980984838164	-1.57537236393662	5.40105350657206
C	5.26310926865815	6.58346993038300	4.63396165809079
C	3.92064690729576	2.75397039867994	0.44858934452347

C	6.31254182173117	3.16996158069056	0.52509004368986
C	7.80868293205788	-2.48231552617442	4.14200621291755
C	5.45120935942691	1.25255145780358	-0.67561161154723
H	5.62677079559718	0.35624428195237	-1.27125961958777
C	8.93625300764128	3.61714818548114	9.05779608529757
H	8.16909635383612	4.21501977949651	9.54986939030311
C	10.88334305528179	2.09122175951115	7.75292249704999
C	4.14800810320927	1.61945947264565	-0.33338818216588
H	3.30360276679777	1.00938067897207	-0.65573110923267
C	10.23598444570331	4.11233496874464	8.91751120483654
H	10.48917462984687	5.09948222192119	9.30556842578847
C	6.61329741813297	6.77944138746992	4.93229010686409
C	11.20884206219755	3.34595511078633	8.27177055081986
H	12.22026151238621	3.73426869654170	8.14820083421704
C	8.60655342184875	2.35950572706473	8.55587067668186
C	4.27134217712444	6.88975129324532	5.56750932412197
C	6.91436074494965	-3.04185682685619	3.22693100734662
H	7.29720528782643	-3.60204191774505	2.37326050411548
C	6.53090336694332	2.02871533107373	-0.24428696250762
H	7.55116295395402	1.74076820282221	-0.49810367302700
C	5.98745335494982	7.58324794056033	7.12779042562547
H	6.27183057930869	7.96328195698277	8.10956429687823
C	5.53929034422267	-2.85996619173462	3.38862892446957
H	4.84407055081674	-3.28286718170863	2.66280934151120
C	5.05532640667471	-2.12578656660318	4.47537320636490
H	3.98359193512392	-1.97059804680069	4.60007010756664
C	4.63891479100114	7.39784476423201	6.81507510477643
H	3.86837252465043	7.62564542523331	7.55223080403646
C	6.97211016789676	7.27300691994016	6.18524485701900
H	8.02683455699193	7.40393814872872	6.42715671778281
H	3.22568108080701	6.69372277223685	5.32832099693400
H	7.36656758597691	6.51215284267662	4.19335338380946
H	11.61963840424911	1.50369076527137	7.20361497265853
H	8.88599159193971	-2.57660637998137	4.00187368946160
H	5.58254772048485	-0.98318939599879	6.24177131491674
H	7.59474183246612	1.96637576930007	8.63695442103235
H	2.91205167054975	3.02540321458265	0.76191734870725
H	7.14193667930760	3.77451435126549	0.88797397778610

$[(IPh)_3Ni_3P_4]$ (**1′ → 1*** transition state)

Ni	13.35602435330095	14.49323448929582	13.15805868699094
Ni	13.27789651026949	12.32136494070568	12.10397037777389
Ni	10.91095281602255	13.36569261837839	12.68278265132808
P	12.42638293220335	12.73755316332853	14.12615189735589
P	12.34460424344972	14.12698886791475	11.23245111257291
P	15.03098277481012	12.88074086453457	13.50702494208402
P	14.98349450673357	13.84693431001975	11.61401178474657
N	8.01834903561892	13.65198915478147	13.24646977020044
N	8.42071836990310	13.66668326238726	11.12063363101300
N	13.29244151686880	10.25059300762158	10.02681337585437
N	13.95236879461364	16.50639009500177	15.15352020529531
N	11.78163929203415	9.84830424663224	11.51322197518138
N	12.03016375550509	16.91195646116720	14.25794308206285
C	10.00762239199934	12.53662813636293	9.65462706261303
C	9.06590713045997	13.54959487923370	9.85795524909363
C	9.06363705190183	13.54833744540653	12.33900887624818
C	9.34828291050941	10.16171141353811	14.93895640627397
C	10.88600227013084	16.90621706477373	13.40783084741458
C	7.04957157265548	13.86029313432332	11.27113844155189
H	6.38357056519895	13.96594042908793	10.42437557088783
C	8.75247775897286	14.44297702786101	8.83083316825230
C	15.49516694485376	15.38108455825201	16.67309991744530
C	6.79544035149993	13.84290514263648	12.60419536531612
H	5.86795112744844	13.97998569831304	13.14400702553790
C	11.05128394447181	16.93676830896579	12.02170881055187
C	9.60799990598796	16.89330050078649	13.97372498494792

C	13.12609486345771	16.07818140651030	14.13662940952615
C	9.58669602827634	10.17863987662535	12.53190576680422
C	14.38558450043453	10.82110942250579	9.29849763284688
C	16.51842565130811	11.90323914012235	7.88062924110016
C	8.78485783942757	10.29405116580371	13.66900080210037
H	7.72375144256854	10.51840106644763	13.55923269151558
C	12.79703367049052	10.73808254018386	11.21778690331498
C	8.16837754363545	13.51890257249855	14.65116310935934
C	14.28235906409455	12.11523139009553	8.78754175241017
C	10.62539881711230	12.41130345946365	8.41155847512979
H	11.35284468811864	11.61486755644464	8.25650009774900
C	11.51882002909320	9.75889771094565	13.93966742191417
C	15.54241395036055	10.06294766995503	9.11146249811891
C	9.36832767169999	13.89661753854001	15.26181609410962
C	15.35639583102710	12.65296936633306	8.07774966884289
H	15.28591575952820	13.67030285870649	7.69253837138244
C	9.38427627598218	14.31721890331839	7.59131896436846
H	9.14918275297067	15.02239397549627	6.79334039830810
C	16.20435902322971	15.94020618724548	14.41750516756536
C	10.71122712529405	9.88763809333122	15.07144629947712
H	11.15807920315753	9.79677150993662	16.06136334726540
C	12.60867639994104	9.11255258759011	9.60355972514255
H	12.85974196730528	8.60285050859630	8.68203868403505
C	7.13152406937076	12.97147729224309	15.41747914245016
C	17.44867356200808	15.36618130925004	14.67213080984717
H	18.19975634396370	15.34337774081493	13.88259767107929
C	9.92689888766203	16.93335959550758	11.19766548679683
H	10.05960827887220	16.93280284579262	10.11584985561322
C	15.23600121429555	15.94263442659164	15.42253998300776
C	10.32060883352390	13.30173450490845	7.37885125535921
C	8.48802088672622	16.89755184057967	13.14086581159267
H	7.49077823407801	16.86740807672593	13.58062545189507
C	16.61087186970266	10.60770743347741	8.39601856285306
H	17.52094614159723	10.02360195067573	8.25548549714988
C	11.65207680930044	8.86422382657457	10.53640498541198
H	10.91066811893651	8.07905200806991	10.61044574977688
C	10.95048317247053	9.91578288339715	12.67622249862779
C	8.64564951746764	16.91330555347749	11.75332916806788
C	17.72243872757405	14.80471518168368	15.92233701444819
C	9.54159944389843	13.69116305525266	16.62950164262456
H	10.49427547004357	13.96204026362746	17.08592377641055
C	16.74678924071236	14.81436059777888	16.92164040653496
H	16.95096300825028	14.36155277043443	17.89244942238359
C	7.30427631745527	12.79429351773100	16.79085792348740
H	6.49690394702232	12.35909862318997	17.38102689122560
C	13.37284914819807	17.52485626797409	15.90392470908173
H	13.89008636669234	17.99488895290055	16.73064152427924
C	12.16386680591238	17.78192781612924	15.34060218831935
H	11.41530223318281	18.52926038605531	15.56965914901757
C	8.51379304260278	13.14354684183317	17.40065818473325
H	6.20725326987075	12.64750980837263	14.93842567184339
H	8.65220109921652	12.98678031120185	18.47101276864249
H	10.16670652107697	14.32905337685194	14.65398610245835
H	10.25968817787867	11.87366752654566	10.47865335330456
H	8.03825007319766	15.24671727911936	9.01185581951619
H	10.81439739976946	13.20714270090507	6.41119170181031
H	17.35822472352321	12.33233687533526	7.33269599832566
H	7.76671520653807	16.90294120997787	11.10789632437167
H	15.96186540891513	16.35376035257120	13.44006884104598
H	12.05621804263032	16.94902844437233	11.60577395727733
H	9.49457979640825	16.84502790952573	15.05658164755358
H	8.72878297486894	10.29579232820443	15.82572163511489
H	14.70899387447138	15.36044218939274	17.42838011541513
H	18.69355228651362	14.34696944892244	16.11362871855891
H	12.58837304459680	9.57586116956228	14.02560974471817
H	13.38246275423924	12.69741380307440	8.97567270742818
H	15.60703830176622	9.06429074761692	9.54493350553746
H	9.16246442706108	10.30935322776460	11.53640888402172

[(IPh)$_3$Ni$_3$P$_6$] (**2′**)

Ni	13.60461323459110	8.54196784608361	16.06698235003657
Ni	14.38106217959208	6.17685342724423	15.86925526319302
Ni	14.11378836737850	7.30774711104430	18.17696119400860
P	15.45680529456928	9.16206616416978	17.19139763574527
P	12.07358322389422	7.01750393998754	15.42943374647581
P	11.95407984450658	7.93050151168315	17.55720616700534
P	12.56171326305027	5.83708159404472	17.20375117074995
P	16.13608045939462	7.08265053584091	17.14706007769775
P	15.66915452464935	8.00127811014225	15.19632163590679
N	12.56339743641803	10.30386078593512	13.92101187830941
N	15.72882668191001	3.53338346712427	16.29719429788791
N	12.99872499888881	11.42131837217254	15.70713608233392
N	14.47786517925872	5.18707164277170	20.26765092316701
N	13.78652227436041	3.29591740838080	15.40823247993337
N	15.93602301775521	6.75370686661648	20.46318605099900
C	14.70993113458522	4.27804808824819	15.73239124883983
C	13.03675149827262	10.13812733634229	15.20509711164955
C	13.31697061546023	4.43841262617986	19.91872905939471
C	12.05386303612915	4.96376095936899	20.19747711301967
C	14.77108387898692	6.44604267276907	19.77731817997591
C	12.42772099762030	9.26737363691894	12.94555377846313
C	13.54733004659769	8.53807408016666	12.54465044949674
C	13.36125600296357	11.80248458571064	17.03646440670323
C	10.91794839012455	4.23783057760691	19.84127995561713
H	9.93003492949518	4.65543627038055	20.03534578235579
C	17.32613709441406	3.95672228307822	18.09233346072941
C	16.96644021823611	4.07583435464661	16.74903559594799
C	17.79892904907775	4.73252345141494	15.84079003118275
C	11.04489281675331	2.99064709063469	19.22407689851046
C	12.24344192037828	11.63304906784310	13.64589000282287
H	11.87075374586882	11.94438292481706	12.67848017453719
C	14.19866350458967	2.02662534922998	15.80286283407509
H	13.60817648009415	1.14174778505943	15.60228981175748
C	12.52623196045107	12.33936634833427	14.76981033899741
H	12.41814870974888	13.39121229004323	15.00142421479899
C	13.45413627591949	3.19864561299690	19.29255460492230
C	11.17490066541094	9.02851163262769	12.37660290990055
C	13.40732667178419	7.55731068425873	11.56311807134585
H	14.28083864576109	6.98267754364451	11.25346017463640
C	15.42663587794042	2.17386768231301	16.36413085452423
H	16.12820574997181	1.44650536873961	16.75306094639055
C	19.00232443351298	5.27715485563654	16.28710271428153
H	19.64348919767021	5.81153050941695	15.58611584070100
C	12.52737940223606	3.54973466927371	14.79404494275271
C	12.46065053364791	4.34597842506335	13.64887481837203
C	11.21823419686460	4.59817308691202	13.06758997172294
H	11.16288451807015	5.24344355762559	12.19264099324977
C	18.53822401786557	4.49188886232743	18.52807680163614
H	18.81538715329360	4.40389658889951	19.57924694320156
C	16.66086225826585	7.96895125765928	20.30536546812228
C	12.69322647926131	11.24395552181153	18.12641681951191
C	10.05501279792983	4.05663349147268	13.61914714321705
C	14.35780554130727	12.76315740832259	17.22131522530254
C	12.16279848418425	7.32497512451816	10.97066192053642
C	11.04703116899408	8.06065968647652	11.37883803957871
H	10.07038592006346	7.87156409314206	10.93207215762494
C	12.31310647306823	2.47217804217315	18.95253113075054
H	12.42150788787821	1.50631194049501	18.45770915073039
C	11.36906616630943	3.01277731036970	15.36056840802174
C	18.03288962734065	7.91939527845155	20.04890374491965
C	10.13199625621607	3.26414372864301	14.76649131876915
H	9.22525632946792	2.85974236524353	15.21763856644879
C	19.37643589306679	5.15361043864385	17.62789468813073
C	15.99120216962095	9.19163741746244	20.38580172162232
C	15.43859180610073	4.72371224007682	21.16557979726368
H	15.34609314052243	3.76712559329028	21.66449130063571
C	16.35965008189918	5.71465583819797	21.28609988980668

H	17.23822358475461	5.79861439053425	21.91301209519053
C	18.74527488582211	9.10789626965262	19.88546073872388
H	19.81324412948474	9.07069076547673	19.66801514648710
C	13.03028374966148	11.65371362495257	19.41609912317737
H	12.51283112851465	11.21346164080256	20.26904011997027
C	14.67416950715811	13.18432547316728	18.51408047748272
H	15.45176483201146	13.93434271444123	18.66241515141359
C	16.71107207785590	10.37352563718023	20.21257562418342
H	16.18474785441462	11.32589965570581	20.24498216790067
C	18.08552202043431	10.33598238667178	19.96742037102744
C	14.01018748534739	12.63111533963994	19.61211877807002
H	12.06247947305757	6.56912168903573	10.19024839959640
H	10.30838032710133	9.58740929720047	12.73081996568122
H	14.51409592259028	8.73915201484516	13.00504218542443
H	20.31680636624152	5.58498277270097	17.97271546955670
H	16.64448083026662	3.48085284878666	18.79666525013311
H	17.47522388251754	4.84030501836412	14.80641305331616
H	18.63878422389154	11.26429341629746	19.82146249252706
H	18.52022704317658	6.95264878169681	19.92985039226149
H	10.15329118323034	2.42884904046924	18.94351406307027
H	9.08646855259703	4.26825739167032	13.16495577884262
H	13.37379291435987	4.78093908748846	13.24431271942225
H	11.44063486887998	2.44693532442049	16.28833401656926
H	14.91496860995511	9.20290621911477	20.55388597135403
H	14.44584552792954	2.82329237692322	19.04158891409173
H	11.97828214638028	5.94917138682378	20.65533346958309
H	14.25684261006375	12.96162152452372	20.62225473034116
H	14.88973738536271	13.15918298089092	16.35590997487093
H	11.92208504542837	10.49277451230195	17.95826487877487

[(IPh)$_2$Ni$_2$P$_5$] (**3′**)

Ni	6.35153355909917	3.51035264259057	3.92821775530809
Ni	7.26827636922801	1.57596381903156	5.49824776115351
P	6.29604726150640	1.23333799145857	3.40146764796528
P	5.02783979569714	1.99453314657066	5.08184432802988
P	6.22225552853812	3.50799171206316	6.25405555570411
P	8.27217218984466	2.27840374933312	3.52833189412974
P	8.22605028279849	3.68976028381575	5.28794720484267
N	9.22374260700717	0.35151347566716	7.32616733163511
N	4.79066258302546	4.65564104339115	1.70912713141137
N	4.90424313584551	6.01328990290763	3.37767702026691
N	8.21822530888984	-1.13805785604697	6.13848072473882
C	9.91063242700632	-0.84462143904014	7.52478348300187
H	10.74679764119922	-0.92336035894489	8.20803776514746
C	3.95652413151919	6.53926605089410	2.50171966840692
H	3.46248568874924	7.48480170912806	2.68635389010003
C	9.27565940444046	-1.78524082166664	6.77529512970270
H	9.44644100687463	-2.84948169866664	6.67348695144428
C	7.31396998289450	-1.75318351696913	5.22464805524593
C	8.17737723753667	0.20548728963900	6.44138428220471
C	3.88493708763850	5.68221011536585	1.44816516903127
H	3.31651187875225	5.72985324908517	0.52794900298524
C	5.42265016004854	4.82021276572375	2.92263742721896
C	9.58227310061523	1.60658276896107	7.89905695131228
C	5.00619761299893	3.52277903695160	0.87162376250794
C	5.93980984838164	-1.57537236393662	5.40105350657206
C	5.26310926865815	6.58346993038300	4.63396165809079
C	3.92064690729576	2.75397039867994	0.44858934452347
C	6.31254182173117	3.16996158069056	0.52509004368986
C	7.80868293205788	-2.48231552617442	4.14200621291755
C	5.45120935942691	1.25255145780358	-0.67561161154723
H	5.62677079559718	0.35624428195237	-1.27125961958777
C	8.93625300764128	3.61714818548114	9.05779608529757
H	8.16909635383612	4.21501977949651	9.54986939030311
C	10.88334305528179	2.09122175951115	7.75292249704999
C	4.14800810320927	1.61945947264565	-0.33338818216588

H	3.30360276679777	1.00938067897207	-0.65573110923267
C	10.23598444570331	4.11233496874464	8.91751120483654
H	10.48917462984687	5.09948222192119	9.30556842578847
C	6.61329741813297	6.77944138746992	4.93229010686409
C	11.20884206219755	3.34595511078633	8.27177055081986
H	12.22026151238621	3.73426869654170	8.14820083421704
C	8.60655342184875	2.35950572706473	8.55587067668186
C	4.27134217712444	6.88975129324532	5.56750932412197
C	6.91436074494965	-3.04185682685619	3.22693100734662
H	7.29720528782643	-3.60204191774505	2.37326050411548
C	6.53090336694332	2.02871533107373	-0.24428696250762
H	7.55116295395402	1.74076820282221	-0.49810367302700
C	5.98745335494982	7.58324794056033	7.12779042562547
H	6.27183057930869	7.96328195698277	8.10956429687823
C	5.53929034422267	-2.85996619173462	3.38862892446957
H	4.84407055081674	-3.28286718170863	2.66280934151120
C	5.05532640667471	-2.12578656660318	4.47537320636490
H	3.98359193512392	-1.97059804680069	4.60007010756664
C	4.63891479100114	7.39784476423201	6.81507510477643
H	3.86837252465043	7.62564542523331	7.55223080403646
C	6.97211016789676	7.27300691994016	6.18524485701900
H	8.02683455699193	7.40393814872872	6.42715671778281
H	3.22568108080701	6.69372277223685	5.32832099693400
H	7.36656758597691	6.51215284267662	4.19335338380946
H	11.61963840424911	1.50369076527137	7.20361497265853
H	8.88599159193971	-2.57660637998137	4.00187368946160
H	5.58254772048485	-0.98318939599879	6.24177131491674
H	7.59474183246612	1.96637576930007	8.63695442103235
H	2.91205167054975	3.02540321458265	0.76191734870725
H	7.14193667930760	3.77451435126549	0.88797397778610

$[(IPh)_3Ni_3P_8]$ (**4′**)

C	15.86196837396580	-1.41078262793282	5.57813802696483
C	13.91566154257561	-1.52600673290628	6.78203925569156
H	13.08929651110481	-1.13816073266393	7.36410766746857
C	14.20422044311361	-2.78725189935525	6.35756180274681
H	13.69096442479797	-3.72846370972299	6.50841446749722
C	15.02941243814228	0.71191985195777	6.47977158577774
C	15.34627791442833	1.22569267020191	7.73768751561384
C	15.50910582538239	2.60394855642685	7.88882621043730
H	15.77201187224979	3.01263800123297	8.86493161554037
C	15.37298686717125	3.45074594678784	6.78581538290343
H	15.52707268769900	4.52403166465747	6.90260813172198
C	15.05953030762538	2.92356504474756	5.53072439198595
H	14.98039964604530	3.57564235392014	4.66107073662418
C	14.87628547720330	1.54983562563474	5.37475368745488
C	16.08305895775317	-3.78790333899501	5.01256951308174
C	17.28527051078770	-4.22943055732107	5.56581325345632
C	18.00495417353359	-5.23370800316600	4.91838353259506
H	18.96437767086223	-5.55168147441463	5.32597078128958
C	17.51280061525072	-5.79997756032897	3.74002869469415
H	18.08336484549862	-6.57731135481298	3.23054565680585
C	16.30136262903903	-5.35887217203977	3.20139685028740
H	15.92636617692627	-5.78650019475729	2.27111562821267
C	15.58402145576022	-4.34153236098911	3.83300484519937
C	22.14956196736879	0.71020012414789	5.87635412222189
C	24.37954512737577	0.33784643086477	6.29902259295839
H	25.24209693037875	-0.22565428026317	6.63227835857639
C	24.26242988825634	1.62148093480538	5.86287727452085
H	25.00363436397862	2.40204558164050	5.74639845769434
C	22.74946960816986	-1.53840829411315	6.64432192475952
C	23.26462565582983	-2.58731539226316	5.88131387141494
C	22.88060835773295	-3.89800234381160	6.17222493906497
H	23.26905592897365	-4.71927238123708	5.56916319327258
C	21.97894796700340	-4.15160103206990	7.20778813368753
H	21.66927934323057	-5.17500158928711	7.42269925648863

C	21.45946789384651	-3.09227988500030	7.95735624021538
H	20.74061846446262	-3.28307990433816	8.75448655434623
C	21.84633345195344	-1.78153636649912	7.68145095370787
C	22.34035942174162	3.02245841520660	5.07423267003168
C	22.91408399235758	3.60480331057039	3.94197719310700
C	22.30334302980193	4.71679871875577	3.35790482296491
H	22.74357101821918	5.16666533592891	2.46716355900686
C	21.12000417203143	5.22988490796360	3.89312733627228
H	20.63858613637650	6.08988452390421	3.42582316951202
C	20.55493233329382	4.64099630686709	5.02846186041554
H	19.62382378376810	5.02577112473353	5.44443345647639
C	21.16729340822188	3.54269983927697	5.62853723055189
C	19.31671526185175	0.33336756725477	0.15614153258124
C	19.90131888088784	1.71079083715298	-1.58892330824907
H	19.86209827046858	2.60517547108481	-2.19809635331846
C	20.60346946373083	0.55450836395729	-1.73735794356979
H	21.28980403697660	0.23566547386797	-2.51153369408051
C	18.28255875668328	2.55795558077590	0.13484603149088
C	18.86659736838827	3.63202792973988	0.80838693043278
C	18.04543952211876	4.57707523079565	1.42564278487716
H	18.49798969670005	5.40288346808000	1.97468776680149
C	16.65644394938960	4.44515744038076	1.36929397601359
H	16.01933290746761	5.18000657253751	1.86283775107224
C	16.08286960094445	3.35876647032247	0.70364563136037
H	14.99950924298336	3.23888359384828	0.67994075575194
C	16.89606746641581	2.40780027839369	0.08639539041485
C	20.79313666803949	-1.55081231630382	-0.39448862325587
C	19.95723164872710	-2.58716148701558	0.03008133466426
C	20.51527771731010	-3.81027390465923	0.39775508501792
H	19.86293022388038	-4.60611231366516	0.75689268053457
C	21.89736974433034	-4.00593846993654	0.32254528759955
H	22.33139944637411	-4.96194910802710	0.61764777781249
C	22.72315934543349	-2.97189675103223	-0.12508391733714
H	23.80314790777018	-3.11536429739009	-0.17637156792529
C	22.17526658001546	-1.73695816559958	-0.47755173677443
N	14.93042974795993	-0.70386553338502	6.29443724214687
N	15.39120949435817	-2.69735732978935	5.63181056591351
N	23.09051106862369	-0.19148733643675	6.31945699290573
N	22.90513850591636	1.83962489679848	5.62970257239910
N	19.11796408513624	1.55235773334709	-0.44792464809495
N	20.22775399803549	-0.27688145658271	-0.68258285356276
P	18.77395360829301	-0.38352844947824	6.58838059526002
P	19.59862792347383	-1.87642940995649	5.12375924605559
P	18.58530479186575	-2.14055742672265	3.07198307979964
P	16.82724639526898	-0.87026647781085	2.50794293158523
P	17.41702215370523	1.17523401185953	3.23913667913362
P	18.37523890476776	1.41700833769066	5.32278604353547
P	19.82644251442610	1.44207062096850	3.30961107431588
P	20.65983846461906	-0.79422403563748	3.21532037608960
Ni	17.42190617432741	-0.77618097029382	4.74719940518499
Ni	20.42301204277537	0.31646785812802	5.18301536123647
Ni	18.90053691482148	-0.11179748992615	1.95645240798967
H	23.80530397993504	3.16011491033143	3.49819136842266
H	20.72802608689189	3.05706518485942	6.49796923873971
H	21.43376673797481	-0.94078233671742	8.23718982918707
H	22.81677649571533	-0.90584900640570	-0.77205161431441
H	15.50049256439546	0.54373035901583	8.57396137632277
H	14.65572086543162	1.11724670875649	4.40018685396652
H	14.66414991851153	-3.94673138066876	3.40178425259686
H	16.47239963038953	1.53390329608760	-0.40751429272022
H	19.95142090670077	3.69344855566639	0.88732866987242
H	23.92578140320175	-2.36708210221906	5.04310837106661
H	18.88479440989488	-2.41356269133135	0.09293316892683
H	17.66611289440625	-3.75694758665421	6.47002572880583

References:

[1] A. J. Arduengo III, S. F. Gamper, J. C. Calabrese, F. Davidson, *J. Am. Chem. Soc.* **1994**, *116*, 4391–4394.

[2] J. B. Diccianni, T. Heitmann, T. Diao, *J. Org. Chem.* **2017**, *82*, 6895–6903.

[3] Y. Hoshimoto, Y. Hayashi, H. Suzuki, M. Ohashi, S. Ogoshi, *Organometallics* **2014**, *33*, 1276–1282.

[4] M. R. Elsby, J. Liu, S. Zhu, L. Hu, G. Huang, S. A. Johnson, *Organometallics* **2018**.

[5] M. R. Elsby, S. A. Johnson, *J. Am. Chem. Soc.* **2017**, *139*, 9401–9407.

[6] a) Sheldrick, G. M. SADABS, Bruker AXS, Madison, USA **2007**; b) CrysAlisPro, Scale3 Abspack, Rigaku Oxford Diffraction **2019**.

[7] R. C. Clark, J. S. Reid, *Acta Cryst. A* **1995**, *51*, 887–897.

[8] G. M. Sheldrick, *Acta Cryst. A* **2015**, *71*, 3–8.

[9] O. V. Dolomanov, L. J. Bourhis, R. J. Gildea, J. A. K. Howard, H. Puschmann, *J .Appl. Crystallogr.* **2009**, *42*, 339–341.

[10] G. M. Sheldrick, *Acta Cryst. C* **2015**, *71*, 3–8.

[11] G. M. Sheldrick, *Acta Cryst. A* **2008**, *64*, 112–122.

[12] R. A. Kendall, H. A. Früchtl, *Theor. Chem. Acc.* **1997**, *97*, 158–163.

[13] F. Weigend, *Phys. Chem. Chem. Phys.* **2002**, *4*, 4285–4291.

[14] F. Neese, F. Wennmohs, A. Hansen, U. Becker, *Chem. Phys.* **2009**, *356*, 98–109.

[15] a) Becke, *Physical Rev. A* **1988**, *38*, 3098–3100; b) Perdew, *Phys. Rev. B* **1986**, *33*, 8822–8824; c) S. Grimme, S. Ehrlich, L. Goerigk, *J. Comput. Chem.* **2011**, *32*, 1456–1465; d) S. Grimme, J. Antony, S. Ehrlich, H. Krieg, *J. Chem. Phys.* **2010**, *132*, 154104; e) F. Weigend, R. Ahlrichs, *Phys. Chem. Chem. Phys.* **2005**, *7*, 3297–3305.

[16] G. Knizia, *J. Chem. Theory Comput.* **2013**, *9*, 4834–4843.
